# Molecular and Electrophysiological Characterization of GABAergic Interneurons Expressing the Transcription Factor COUP-TFII in the Adult Human Temporal Cortex

**DOI:** 10.1093/cercor/bhv045

**Published:** 2015-03-18

**Authors:** Csaba Varga, Gabor Tamas, Pal Barzo, Szabolcs Olah, Peter Somogyi

**Affiliations:** 1 Research Group for Cortical Microcircuits of the Hungarian Academy of Science, Department of Physiology, Anatomy and Neuroscience; 2 Department of Neurosurgery, University of Szeged, Szeged, Hungary; 3 MRC, Brain Networks Dynamics Unit, Department of Pharmacology, University of Oxford, Oxford OX1 3TH, UK; 4 Institute of Experimental Medicine, Hungarian Academy of Sciences, Budapest, Hungary; 5 Current address: Szentágothai Research Centre, Department of Physiology, University of Pécs, Pécs, Hungary

**Keywords:** calretinin, dendritic synapses, inhibition, neuropeptides, pyramidal cell

## Abstract

Transcription factors contribute to the differentiation of cortical neurons, orchestrate specific interneuronal circuits, and define synaptic relationships. We have investigated neurons expressing chicken ovalbumin upstream promoter transcription factor II (COUP-TFII), which plays a role in the migration of GABAergic neurons. Whole-cell, patch-clamp recording in vitro combined with colocalization of molecular cell markers in the adult cortex differentiates distinct interneurons. The majority of strongly COUP-TFII-expressing neurons were in layers I–III. Most calretinin (CR) and/or cholecystokinin- (CCK) and/or reelin-positive interneurons were also COUP-TFII-positive. CR-, CCK-, or reelin-positive neurons formed 80%, 20%, or 17% of COUP-TFII-positive interneurons, respectively. About half of COUP-TFII-/CCK-positive interneurons were CR-positive, a quarter of them reelin-positive, but none expressed both. Interneurons positive for COUP-TFII fired irregular, accommodating and adapting trains of action potentials (APs) and innervated mostly small dendritic shafts and rarely spines or somata. Paired recording showed that a calretinin-/COUP-TFII-positive interneuron elicited inhibitory postsynaptic potentials (IPSPs) in a reciprocally connected pyramidal cell. Calbindin, somatostatin, or parvalbumin-immunoreactive interneurons and most pyramidal cells express no immunohistochemically detectable COUP-TFII. In layers V and VI, some pyramidal cells expressed a low level of COUP-TFII in the nucleus. In conclusion, COUP-TFII is expressed in a diverse subset of GABAergic interneurons predominantly innervating small dendritic shafts originating from both interneurons and pyramidal cells.

## Introduction

In spite of the pioneering Golgi studies of Ramon y Cajal (1899) and some recent studies (Kisvarday et al. 1986, 1990; DeFelipe 1997; del Rio and DeFelipe 1997a; Alkondon et al. 2000; Szabadics et al. 2006; Molnar et al. 2008), the analysis of neuronal circuits in the human cerebral cortex has lagged behind that in other mammals, largely due to the lack of suitable tissue. A particular challenge in human and other animal cortices is the identification of the highly diverse GABAergic neuronal types, and the definition of homologies between cell types in the human and the extensively investigated rodent cortex. An informative approach is to compare the molecular expression profiles of different neurons together with their axonal and dendritic patterns, a method that has proved revealing in some cases (Freund and Buzsaki 1996; Kawaguchi and Kubota 1997; Toledo-Rodriguez et al. 2004, 2005; Packer et al. 2013). Although species-to-species variability in the expression of signaling molecules has been described (DeFelipe 1997; Xu et al. 2006; Cauli et al. 2014), there are also highly conserved patterns of consistent molecular characteristics that correlate with synaptic connections of a given cell type. For example, a subpopulation of soma-innervating basket cells expresses the calcium-binding protein parvalbumin in all cortical areas of all species. Molecular and physiological diversity among cortical GABAergic interneurons is controlled by the action of sets of transcription factors resulting in exquisitely specific synaptic circuitry. Developmental abnormalities lead to psychiatric conditions (Rubenstein and Merzenich 2003; Marin 2012; Karayannis et al. 2014; Lewis 2014).

Transcription factors are promising markers of cell identity in both the adult and the developing cortex (Letinic et al. 2002; Rakic and Zecevic 2003; Cobos et al. 2006; Liodis et al. 2007; Miyoshi et al. 2013; Kessaris et al. 2014; Marin and Muller 2014). Cortical GABAergic interneurons are generated in the telencephalic neuroepithelium of the subpallium and migrate to the cortical mantle under the control of transcription factors such as Dlx1, 2, and 5, Arx, Lhx6, Cux2, NPAS1, MafB, and COUP-TFII [Tripodi et al. 2004; for review, see Wonders and Anderson (2006); Miyoshi et al. (2013)]. Different areas in the ganglionic eminences are delineated by the expression of partially distinct transcription factors (Nery et al. 2002; Xu et al. 2004; Flames and Marin 2005; Vucurovic et al. 2010; Kessaris et al. 2014). There are indications that neurons generated in different parts of the ganglionic eminences become different interneuronal types, that is, they end up occupying distinct synaptic and activity-related temporal positions in the cortical network (Nery et al. 2002). Some transcription factors involved in development are also retained in adult cortical neurons (Cobos et al. 2006; Ma et al. 2012, 2013) and may continue to contribute to their circuit positions. In the mouse, a given transcription factor is expressed only in some types of GABAergic neuron as revealed by the expression of calcium-binding proteins and neuropeptides (Alcantara et al. 1998; Erbel-Sieler et al. 2004; Cobos et al. 2005). However, these widely used molecular cell markers do not identify an interneuron type in terms of connectivity, as all of them are expressed in cells with distinct spatio-temporal and synaptic features. With few exceptions, only a combination of expressed molecules can delineate and define a cell type (Cauli et al. 2000; Monyer and Markram 2004; Toledo-Rodriguez et al. 2005), and further molecular combinations, including transcription factors, in both animal and human cortical neurons may help to define neuronal phenotypes.

The steroid/thyroid hormone receptor superfamily of proteins includes chicken ovalbumin upstream promoter transcription factors I and II (COUP-TFs, II is also known as Nr2f2), and these are expressed in the dorsal medial ganglionic eminence and the caudal ganglionic eminence (CGE), as well as in migratory and adult interneurons, and have been shown to be necessary for interneuronal adhesion (Tripodi et al. 2004) and development (Tsai and Tsai 1997; Alfano et al. 2014) in the mouse. Originally recognized as transcriptional activators for the chicken ovalbumin gene, COUP-TFs have been shown to be repressors and activators of several genes (Tsai and Tsai 1997; Alfano et al. 2014), and COUP-TFII is widely expressed during organogenesis. High levels of this protein occur in the salivary gland, the lung and esophagus, the stomach, the pancreas primordium, the prostate, and the kidney (Tsai and Tsai 1997), and it is expressed in some cells of the blood vessels (You et al. 2005). During neurogenesis, embryonic Cajal–Retzius cells, which play a crucial role in guiding migrating cortical interneurons, contain COUP-TFII (Tripodi et al. 2004). In both the rodent and human cerebral cortex, a large fraction of interneurons derived from the dorsal lateral ganglionic eminence (dLGE) and the CGE (Ma et al. 2012, 2013). In the human brain, COUP-TFII expression was also described in the subventricular proliferative zones (Reinchisi et al. 2012) of the dorsal pallium. COUP-TFII is also expressed in interneurons of the adult rodent hippocampus where the cell types and their contribution to network activity have been explored (Fuentealba et al. 2010). To characterize human cortical neurons that express COUP-TFII and to test if the presence of this transcription factor is predictive of cell type identity, we used human cortical biopsies for whole-cell, patch-clamp recording in vitro combined with imunohistochemical characterization of the recorded cells and neuronal populations in immunohistochemically colocalization experiments.

## Materials and Methods

All procedures concerning patients were performed with the approval of the University of Szeged and in accordance with the Declaration of Helsinki. Human cortical brain slices were obtained from association cortices. Patients were diagnosed with deep brain tumors, and written informed consent was obtained prior to surgery (aged 18–72 years *n* = 10, 7 males and 3 females; Table 1). Samples were taken from sites at least 1.5 cm from the edge of the tumor mass. Cortical tissue at the immediate vicinity of the area used for experiments underwent neuropathological examination, and samples showing pathological alterations were not included in this study. Anesthesia was induced with intravenous midazolam and fentanyl (0.03 mg/kg, 1–2 μg/kg, respectively). A single dose of propofol (1–2 mg/kg) was administered intravenously. To facilitate endotracheal intubation, the patient received 0.5 mg/kg rocuronium. After 2 min, the trachea was intubated and the patient was ventilated with a mixture of O_2_–N_2_O at a ratio of 1 : 2. Anesthesia was maintained with sevoflurane at a minimal alveolar concentration volume of 1.2–1.5. Blocks of healthy tissues were removed from medial or inferior parts of the gyrus temporalis, and incubated in oxygenated cold Ca^2+^-free artificial cerebrospinal fluid. Cortical slices were prepared at 350 μm thickness as described previously (Szabadics et al. 2006), and the remaining blocks of tissue were immersed in a fixative containing 4% paraformaldehyde and approximately 0.2% (w/v) picric acid dissolved in 0.1 M PB pH 7.2–7.4, for 4–10 h for immunohistochemical experiments.

**Table 1:** Origin and location of biopsies

Patient code	Sex	Age (years)	Cortical area
1	Male	37	Left gyrus temporalis inferior, middle third
2	Male	72	Left gyrus temporalis medialis, middle third
3^a^	Male	48	Left gyrus temporalis inferior, middle third
4^a^	Male	36	Right gyrus temporalis inferior, middle third
5^a^	Female	18	Right gyrus temporalis medialis, middle third
6	Female	49	Left gyrus temporalis medialis, middle third
7	Male	54	Right gyrus temporalis medialis, middle third
8	Male	46	Right gyrus temporalis medialis, middle third
9	Male	54	Right gyrus temporalis medialis, middle third
10	Female	71	Left gyrus temporalis medialis, posterior third

^a^Specimens used for neuronal population quantification.

### Electrophysiology

Cortical slices were incubated at room temperature for 1 h in a solution composed of (in mM) 130 NaCl, 3.5 KCl, 1 NaH_2_PO_4_, 24 NaHCO_3_, 1 CaCl_2_, 3 MgSO_4_, and 10 D(+)-glucose, saturated with 95% O_2_ and 5% CO_2_. The solution used during recordings differed only in that it contained 3 mM CaCl_2_ and 1.5 mM MgSO_4_. Recordings were obtained at approximately 35 °C from neurons visualized by infrared differential interference contrast videomicroscopy using a BX60WI microscope (Olympus, Tokyo, Japan), a Hamamatsu CCD camera (Bridgewater, NJ, USA), Luigs & Neumann infra-patch set-up (Ratingen, Germany), and a HEKA Elektronik EPC 10/double patch-clamp amplifier (Lambrecht/Pfalz, Germany). Micropipettes (5–7 MΩ) were filled with (in mM) 126 K-gluconate, 4 KCl, 4 ATP-Mg, 0.3 GTP-Na_2_, 10 HEPES, 10 creatine phosphate, and 8 biocytin at pH 7.25 at 300 total mOsm. Signals were filtered at 5 kHz, digitized at 10 kHz, and analyzed with the PULSE software (HEKA Elektronik). At the end of the recording, special care was taken not to remove the cell nucleus with the patch electrode. Intrinsic membrane properties (resting membrane potentials, input resistance, time constant, sag, and rebound) and firing parameters (AP amplitude, half width, threshold, afterhyperpolarization amplitude, and firing pattern in response to rheobasic and larger amplitude current injections) of the recorded cells were analyzed with the PULSE software (HEKA Elektronik). Membrane potential values were corrected by the junction potential, which was 13.74 mV. Measurements could be taken from 15 of the 17 biocytin-labeled cells that were immunopositive for COUP-TFII. The quality of recording was suboptimal for one cell, but it was recovered for anatomical analysis.

### Immunohistochemistry of Biocytin-Labeled Cells

Following recording, the slices were immersed in a fixative containing 4% paraformaldehyde and approximately 0.2% (w/v) picric acid dissolved in 0.1 M PB pH 7.2–7.4, for 4–6 h at 4 °C, and resectioned at 60 μm thickness. Following washing in PB, the recorded cells were first visualized with overnight incubation in Alexa-488-conjugated streptavidin (Molecular Probes, Leiden, The Netherlands), diluted 1 : 1000 in 0.1 M PB containing 0.05% NaN_3_. After examination by epifluorescence microscopy, the sections containing the somata of the labeled neurons were incubated in 5% normal horse serum and diluted in 0.1 M PB (blocking buffer containing 0.05% NaN_3_) to block nonspecific antibody-binding sites. Thereafter, the sections were incubated in mouse-anti-COUP-TFII (1 : 1000, Perseus Proteomics, Inc., Tokyo, Japan) dissolved in a blocking buffer. After several washes in 0.1 M PB, the immunoreactions were visualized with Cy3- or Cy5-conjugated donkey-anti-mouse antibodies (1 : 500, Jackson Immunoresearch, West Grove, PA, USA). Following evaluation of the immunoreaction, the sections were further incubated in a mixture of primary antibodies containing goat-anti-calretinin (1 : 1000, Swant, Bellinzona, Switzerland) and rabbit-anti-pro-cholecystokinin (CCK) (1 : 2000, gift from Andrea Varro, Liverpool University). After an extensive wash in 0.1 M PB, the sections were further incubated in a mixture of Alexa350-conjugated donkey-anti-sheep (1 : 250, Molecular Probes) and Cy3- or Cy5-conjugated donkey-anti-rabbit antibodies (1 : 500, Jackson Immunoresearch). We have used donkey-anti-sheep secondary antibody for the detection of the antibody to calretinin raised in goat, as the secondary antibody cross-reacted with both goat and sheep antibodies and produced a clear signal. All antibody incubations were conducted for approximately 16–20 h at room temperature. To reduce the high autofluorescence of lipofuscin in the fixed tissue sections, additional Sudan Black B (Sigma-Aldrich, Dorset, UK) treatment was performed (see below).

The sections were mounted on slides in Vectashield (Vector Laboratories, Burlingame, CA, USA). After photography, the sections were demounted, washed in PB, and biocytin was visualized with the avidin-biotinylated horseradish peroxidase method (ABC kit, Vector Laboratories) using 3-3′-diaminobenzidine (DAB, Sigma-Aldrich) as a chromogen as described previously (Buhl et al. 1997). Three-dimensional light microscopic reconstructions of dendritic and axonal fields were produced using Neurolucida (MicroBrightField, Williston, VT, USA) and a ×100 objective. Areas rich in axons from selected neurons were further processed for electron microscopic analysis of synaptic targets using serial sections mounted on single-slot Pioloform-coated copper grids. Axonal profiles filled by biocytin were followed and tilted as necessary to reveal synaptic junctions, photographed with a CCD camera, and the largest linear extent of the synaptic junction was measured.

### Multiple Immunofluorescence Labeling of Neuronal Populations

The samples containing all layers and some white matter were taken from the top of gyro from 3 patients (Table 1). Immersion-fixed human cortical slices were used for testing the molecular expression profiles of neurons. Blocks of tissue were immersed in a fixative containing 4% paraformaldehyde and approximately 0.2% (w/v) picric acid dissolved in 0.1 M PB for 5–10 h. The blocks were then washed in 0.1 M PB and sectioned with a vibratome at 60 μm thickness. All immunoreactions were performed on free-floating sections. Endogenous peroxidase activity and lipofuscin autofluorescence were reduced by treatment with 1% H_2_O_2_ in PB for 20 min, followed by incubation in 70% ethanol for 5 min, a rinse in 1% Sudan Black B (Sigma-Aldrich) dissolved in 70% ethanol, for 10 min, and finally 70% ethanol for 5 min. Then, the sections were incubated for 1 h in a blocking buffer, followed by mouse-anti-reelin antibody (1 : 50 000, 142CL Chemicon International, Temecula, CA, USA) dissolved in blocking buffer, overnight. After several washes in PB, the sections were incubated in biotinylated donkey-anti-mouse antibody (1 : 500, Jackson Immunoresearch) dissolved in blocking buffer overnight, followed by avidin-biotinylated horseradish peroxidase complex (Vectastain ABC kit, Vector Laboratories) for 2 h, and finally in Cy3-conjugated tyramide (conjugated in house) was used as a chromogen. The peroxidase reaction was carried out for 2–4 h using 0.006% H_2_O_2_ as substrate, followed by washing the sections in 0.1 M PB.

After a short microscopic examination of the quality of the immunoreaction, the sections were further incubated in mouse-anti-COUP-TFII antibody (1 : 1000) dissolved in a blocking buffer, washed in 0.1 M PB, and then incubated in Alexa488-conjugated donkey-anti-mouse antibody (1 : 1000, Molecular Probes). The sections already labeled for reelin and COUP-TFII were further incubated in a mixture of rabbit-anti-CCK antibody (1 : 2000) and goat-anti-calretinin antibody (1 : 1000). After several washes in 0.1 M PB, the sections were incubated in a mixture of Cy5-conjugated donkey-anti-rabbit antibody (1 : 500, Jackson Immunoresearch) and Alexa350-conjugated donkey-anti-goat antibody (1 : 500, Molecular Probes). After photography, the sections were demounted and incubated in mouse-anti-SMI311 antibody (1 : 1000, overnight, Cambridge Biosciences, Berkeley, CA, USA) for the detection of non-phosphorylated neurofilament protein (NPNFP), visualized by A488-coupled donkey-anti-mouse secondary antibody. This last immunolabeling was used to delineate the borders of cortical layers, as it is highly expressed in some pyramidal cells.

Additional sections from the same specimens were incubated in a mixture of mouse-anti-COUP-TFII, goat-anti-parvalbumin (Swant), rat-anti-somatostatin (Chemicon International), and rabbit-anti-calbindin (Swant) antibodies (all at 1 : 1000 dilution) overnight, washed extensively, and incubated in a mixture of secondary antibodies that included A350-coupled donkey-anti-goat (Molecular Probes), A488-coupled donkey-anti-mouse, Cy3-coupled donkey-anti-rat, and Cy5-coupled donkey-anti-rabbit antibodies (Jackson Immunoresearch) dissolved in blocking buffer. All sections were mounted in Vectashield (Vector Laboratories) on slides.

### Controls

Characterization of the primary antibodies has been previously published or provided by the manufacturer (Table 2). The experiments were performed with highly cross-absorbed species-specific secondary antibodies from Jackson Immunoresearch or Molecular Probes. To control the possible cross-reactivity between secondary antibodies with multiple primary antibodies, control sections were treated with the previously described protocols, but only one primary antibody was applied, followed by all the secondary antibodies. This control was performed for all antibodies separately. No false reactivity was detected. The highest risk for cross-labeling was between mouse-anti-reelin and mouse-anti-COUP-TFII antibodies, because these were raised in the same host species. However, the 2 molecules showed different subcellular localization when detected alone. In this case, the mouse-anti-reelin antibody was applied with the highest possible dilution (1 : 50 000). As a test, after signal amplification of the immunolabeling with Cy3-tyramide, the original protocol was carried out, but the second mouse primary antibody (to COUP-TFII) was replaced by 10% normal mouse serum to test for the possible capture of the second mouse primary antibody by biotinylated donkey-anti-mouse IgG. The control sections were washed in 0.1 M PB and finally incubated overnight in Alexa488-conjugated donkey-anti-mouse IgG. The first primary antibody concentration was far below the detection threshold of this last secondary antibody, and no signal was detected in the Alexa488 fluorescent channel. Tests were also carried out using much longer camera exposure times than usual, but no Alexa-488 signal could be detected.

**Table 2:** Use and characteristics of primary antibodies

Primary antibody to	Raised in species	Code	Dilution	Protein conc. original	Source	Characterization, reference, or test	Immunogen
Calbindin	Rabbit	CB-38	1 : 1000		Swant, Bellizona, Switzerland	Labeling pattern in the rat as published with other antibodies	Rat recombinant calbindin
Calretinin (CR)	Goat	CG1	1 : 1000		Swant, Bellizona, Switzerland	Schwaller et al. (1999), western blot, brain, and cell lines expressing recombinant CR	Recombinant human calretinin
COUP-TFII	Mouse	PP-H7147-00, 2ZH7147H	1 : 1000	1 mg/mL	Perseus Proteomics, Inc./R&D Systems	Qin et al. (2007), tested in gene deleted tissue	Recombinant human COUP-TFII
NPNFP, non-phosphorylated neurofilament protein	Mouse	SMI-311R	1 : 1000		Convance/Cambridge Biosciences	Mixture of several monoclonal antibodies recognizing NF-H and NF-M subunits. Originally developed by Sternberger Monoclonals, Inc.	Brain
Parvalbumin	Goat	PVG 214	1 : 1000		Swant, Bellizona, Switzerland	Labeling pattern in the rat as published with other antibodies	Rat muscle parvalbumin
Pro-CCK^a^	Rabbit		1 : 2000		A. Varro, Liverpool University	Morino et al. (1994)	Residues 107–115 of rat pro-CCK
Reelin	Mouse	MAB5366	1 : 50000	1 mg/mL	Chemicon	de Bergeyck et al. (1998), immunoblot	Recombinant mouse reelin residues 40–189
Somatostatin	Rat	MAB354	1 : 200		Chemicon	Labeling pattern in the rat as published with other antibodies	Synthetic cyclic somatostatin 1–14

^a^This antibody labels the Golgi apparatus in the soma where pro-CCK is processed and packaged for transport, and for simplicity the signal obtained is described in the text as CCK.

### Image Acquisition and Quantification of Co-labeling

For quantitative analysis, images were collected with an Olympus epifluorescence microscope with ×20, 0.5 numerical aperture UPlanFI lens and a SPOT 7.4 slider CCD camera and appropriate filters. From each field of view, 5 equally spaced focal depths were recorded in all fluorescence channels, spanning from the top to the bottom of the 60-μm thick section. Cells having an ambiguous colocalization of signals were also recorded with a ×40, 0.75 numerical aperture UPlanFI lens. Brightness and contrast were adjusted for the whole frames in Photoshop. The images were merged in each channel separately to survey the cortex spanning the layers from the pia to the white matter, and the position of cells positive for at least one of the markers was numbered and entered into a spreadsheet.

Data are presented as mean ± standard deviation.

## Results

### Distribution and Characterization of COUP-TFII-Immunoreactive Cells

**Figure 1.**
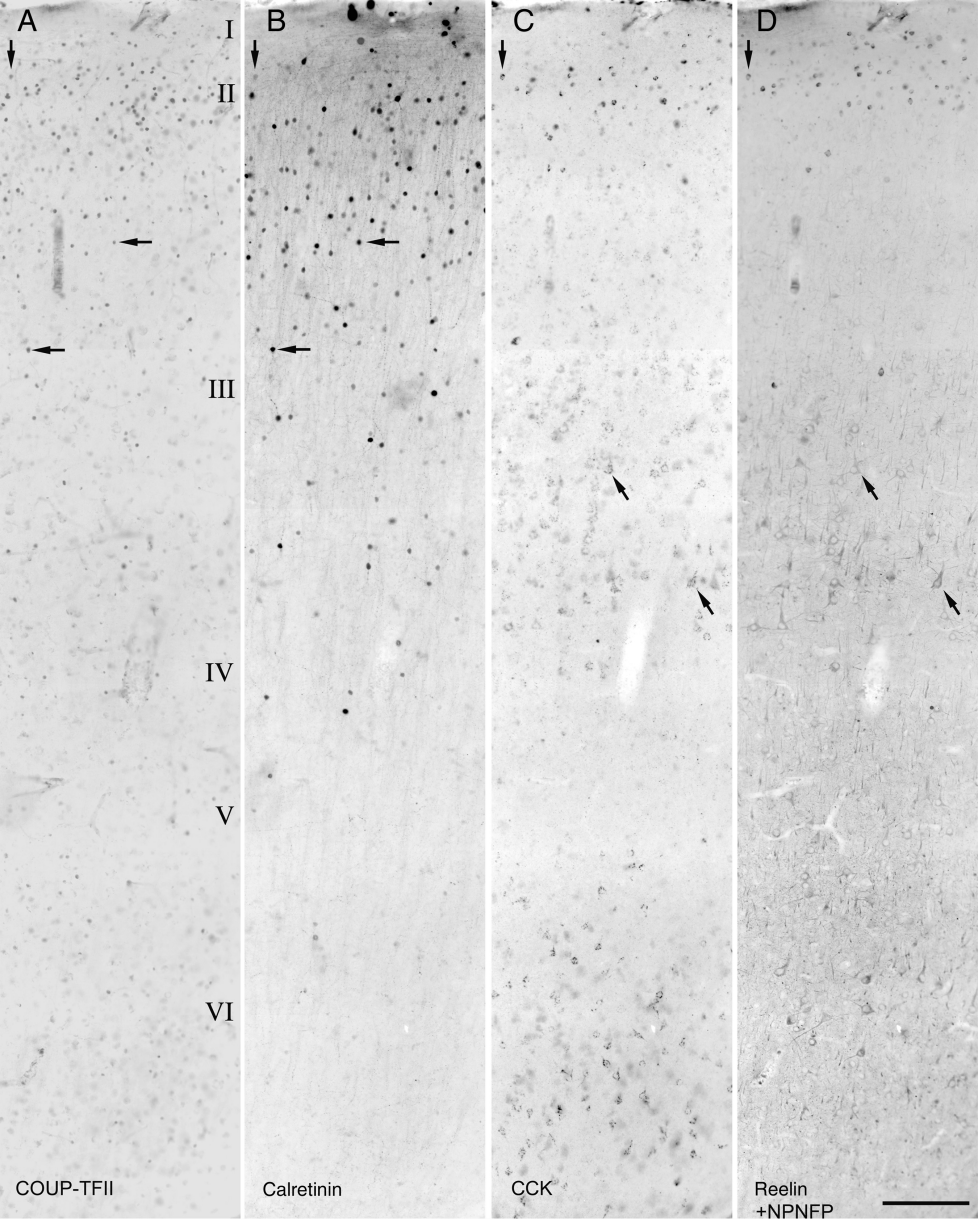
Radial distribution of COUP-TFII- (*A*), calretinin- (*B*), CCK- (*C*), reelin-, or NPNFP- (*D*) immunoreactive cells in the adult human temporal cortex. Reelin and NPNFP immunoreactivity could be visualized in the same fluorochrome because they label distinct cells in different layers. The original dark-field fluorescent images were inverted for better visibility. COUP-TFII-positive cell nuclei are mostly situated in supragranular layers, whereas small calretinin-, CCK-, and/or reelin-positive interneurons are most frequent. Examples of cells labeled for at least 2 molecules are marked by arrows. In addition to the strongly CCK-immunoreactive small interneurons, some pyramidal cells in lower layer III and layer VI are also CCK-positive. In (*D*), antibodies to reelin reveal small interneurons mainly in layers I and II, whereas NPNFP reveals large pyramidal cells, which aid the delineation of laminar boundaries. Scale bar: 200 µm.

**Figure 2.**
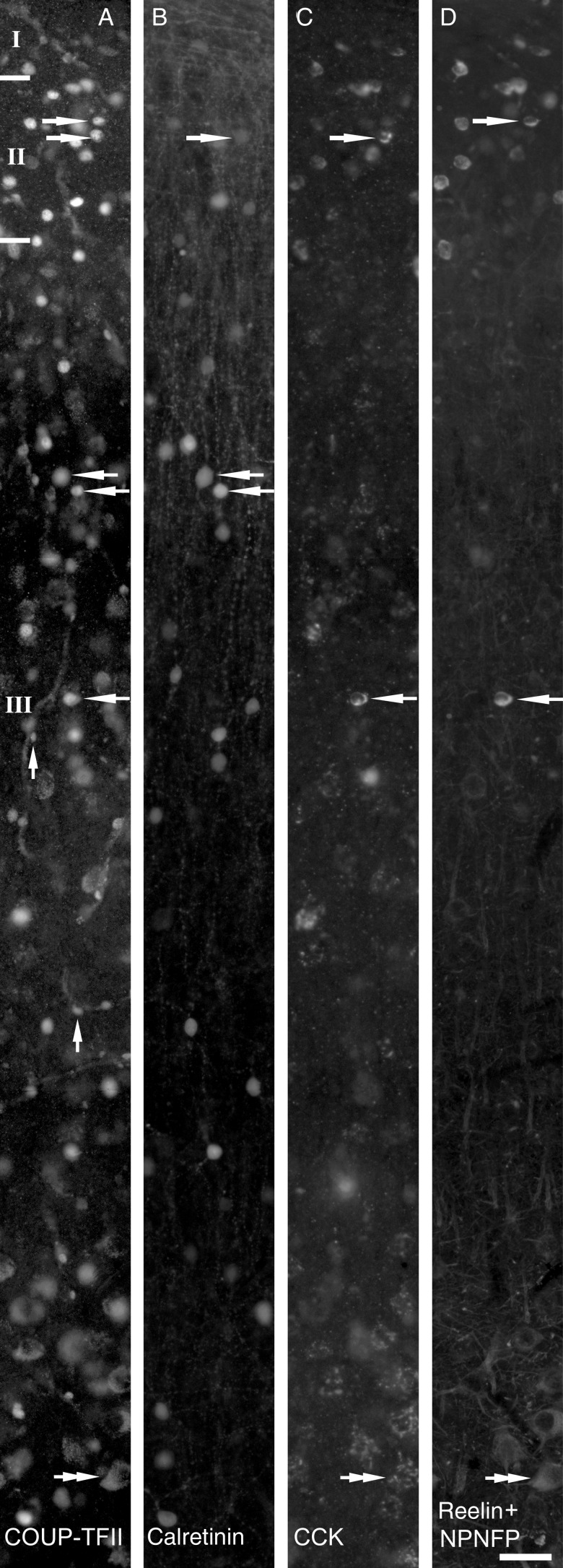
Part of the section shown in Figure 1 is shown at high magnification as immunofluorescence images using 4 filter combinations and demonstrating the distribution of COUP-TFII- (*A*, Alexa488), calretinin- (*B*, Alexa350), CCK- (*C*, cy5), reelin-, and NPNFP- (*D*, cy3) immunopositive neurons in the supragranular layers. Examples of the colocalization of 2 or more immunoreactivities are indicated by arrows. Labeling for COUP-TFII shows small bright nuclei of some interneurons, as well as very small nuclei in the wall of 2 blood vessels (*A*, vertical arrows). In the Alexa488 channel, in lower layer III, large pyramidal cells show clearly distinguishable cytoplasmic lipofuscin autofluorescence (*A*, double arrow). Large pyramidal cells show weak, patchy pro-CCK immunoreactivity present in the perinuclear Golgi apparatus (*C*, double arrow) and reactivity for NPNFP (*D*, double arrow) in the soma and proximal dendrites. Scale bar: 40 µm.

**Figure 3.**
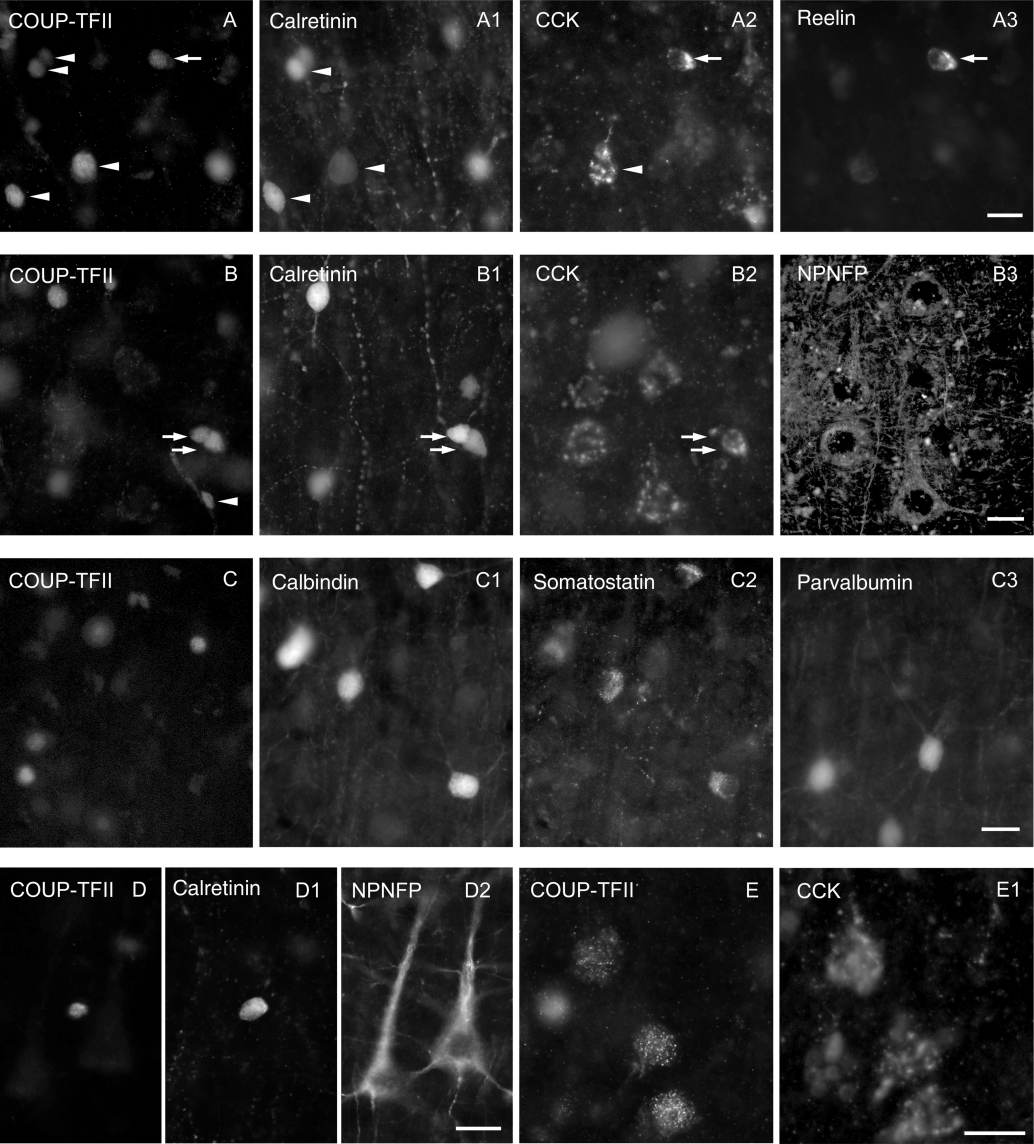
Immunofluorescence characterization of COUP-TFII expressing using up to 5 antibodies and 4 filter sets on the same cells. (*A*) Most calretinin-positive neurons (e.g., arrowhead) are COUP-TFII-positive and some of them express CCK (arrowhead) and/or reelin (arrow) in upper layer II. (*B*) In middle layer III, most calretinin-positive neurons are also COUP-TFII-positive (arrows) and some express CCK (lower arrow). Many pyramidal cells also express CCK in patches representing the Golgi apparatus (*B2*) and these cells are also immunopositive for NPNFP (*B3*), but immunonegative for COUP-TFII and calretinin. The small nuclei of some cells in the wall of blood vessels (*B*, arrowhead) are immunopositive only for COUP-TFII. (*C*) Interneurons positive for calbindin, somatostatin, or parvalbumin were always immunonegative for COUP-TFII in layers II and III. (*D*) A calretinin-positive interneuron is also positive for COUP-TFII, but nearby NPNFP-positive pyramidal cells are immunonegative for COUP-TFII in lower layer III. (*E*) Weakly COUP-TFII-immunopositive large nuclei belong to CCK-positive pyramidal cells in layer VI. The CCK-positive perinuclear patches represent the Golgi apparatus. Scale bar: *A*–*E*, 20 µm.

Immunoreactivity for COUP-TFII was located in the nuclei of 3 distinct types of cell (Figs 1–3). Most conspicuous were medium diameter (8.5 ± 1.1 μm, *n* = 20), strongly immunopositive nuclei mainly in layers I, II, and upper III, and much less frequently in all other layers. Very small, strongly positive nuclei, often of an elongated shape (short axis, 4.0 ± 0.6 μm; long axis 6.7 ± 0.9 μm, *n* = 31), were seen around blood vessels (Fig. 2*A*). Finally, large (12.9 ± 1.5, *n* = 21) weakly positive nuclei were present mostly in layer VI (Fig. 3*E*) and less frequently in layer V.

The distribution of medium size strongly positive nuclei corresponded to previously published patterns of calretinin-positive interneurons, which are thought to be GABAergic neurons (DeFelipe 1997; del Rio and DeFelipe 1997b; Gabbott, Dickie, et al. 1997; Gabbott, Jays, et al. 1997; Meskenaite 1997). Therefore, we tested the degree of overlap of these molecules with CCK that has previously been shown to coexist with calretinin in some interneurons of the rat (Kawaguchi and Kubota 1997). In addition, layer I had a significant number of COUP-TFII-positive nuclei and this layer contains neurons expressing reelin in several species (Alcantara et al. 1998; Pesold et al. 1998; Martinez-Cerdeno and Clasca 2002; Pohlkamp et al. 2014), a signaling molecule known to be co-expressed with COUP-TFII during development (Tripodi et al. 2004). Therefore, we tested the degree of co-expression of these 2 molecules as well. To maximize the information, we carried out the tests on the same sections from 3 patients using antibodies raised in 3 different species and also used the tyramide intensification method to include sequentially 2 mouse monoclonal antibodies. Because the antibody to CCK revealed a significant population of pyramidal-like cells, we included a fifth antibody, raised in mouse to NPNFP, to characterize these cells.

**Figure 4.**
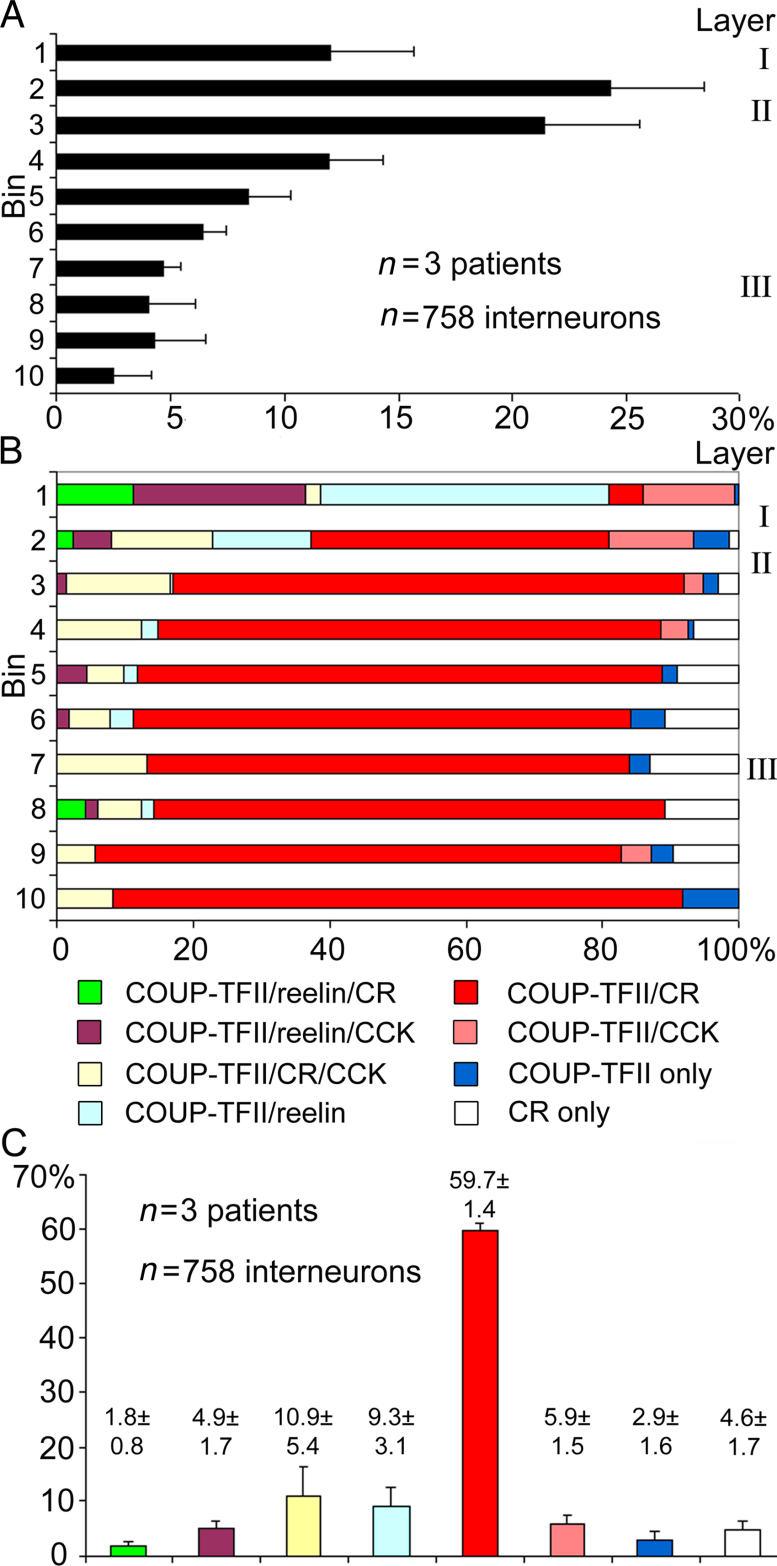
Distribution of strongly COUP-TFII-positive interneurons and colocalization patterns with calretinin, reelin, and CCK displayed in 10 radial bins from the pia to the bottom of layer III. Pyramidal cells positive for CCK were excluded. (*A*) Average (±SD) proportions of COUP-TFII-positive cells per bin. (*B*) Depth profile of the colocalization distribution of the 4 molecules shown as average of the 3 patients. Most COUP-TFII-positive cells were positive for at least one of the 3 molecules. All reelin-positive cells were COUP-TFII-positive and mostly located in layers I and upper II. Few calretinin-positive cells were COUP-TFII-negative. (*C*) Distribution of cells (mean ± SD) showing different colocalization patterns summed from all supragranular layers. Most COUP-TFII-positive cells are immunopositive for calretinin, and/or CCK, and/or reelin, and those immunonegative for all 3 molecules represent on average only 2.9% of cells. Note the small variation between the 3 individuals. Colour-coding of diagrams in (*B*) and (*C*) is identical.

We could not detect differences between the 3 patients. Immunoreactivity for COUP-TFII in strongly positive neuronal nuclei and calretinin-positive interneurons showed very similar laminar distributions (Fig. 1*A*,*B*). Calretinin immunoreactivity was present throughout the cytoplasm, including the dendrites and axons. Strongly CCK-immunopositive medium-sized cells were mostly in layers I, II, and upper III, whereas much larger more weakly positive pyramidal-shaped cells were present in lower layer III and layer VI (see below). Indeed, labeling for NPNFP showed that these weakly CCK-positive large cells are pyramidal cells (Figs 1*C*,*D* and 3*E*). Reelin-positive cells were mainly in layers I and II. Co-expression of immunoreactivity for COUP-TFII and calretinin and/or CCK and/or reelin was extensive in all layers. However, most of the strongly COUP-TFII-positive medium-sized nuclei identifying putative interneurons were in the supragranular layers in all 3 patients. Indeed, of the total number of interneurons, immunopositive for CCK (strong), calretinin, or reelin, 98.2 ± 0.3%, 95.2 ± 1.6%, and 97.4 ± 2.3% were situated in the supragranular layers with a combined thickness of 1130 ± 90 μm (*n* = 3). Therefore, we have restricted the detailed examination of the co-expression of 4 molecules to interneurons in layers I–III (total *n* = 765 cells; Fig. 4). The combinations of colocalized of calretinin, reelin, and CCK with COUP-TFII resulted in 11 categories of neurons. Three of these categories representing only 7 cells, together formed <0.5% of the total population, were not considered further. The distribution of the remaining 758 neurons (patient 1, *n* = 274; patient 2, *n* = 190; patient 3, *n* = 294) are shown in Figure 4 in 8 categories. Cells were counted in a radial 590-μm wide strip from each of 3 patients. The distance between the pia and the bottom of layer III was divided into 10 equal bins, and all neurons labeled for at least one of the 4 molecules were counted. Calretinin- and/or CCK- and/or reelin-positive interneurons constituted 97 ± 1.6% of COUP-TFII-positive interneurons in the supragranular layers. Most calretinin- and/or CCK-positive interneurons were COUP-TFII-positive. Calretinin- and CCK-positive interneurons formed 75.8 ± 5.0% and 22.7 ± 2.0% of COUP-TFII-positive cells, respectively, in layers I–III. About half of the CCK-positive interneurons were also calretinin-positive, but only 13.9 ± 6.1% of calretinin-expressing cells were CCK-positive.

Reelin-positive interneurons constituted 16.9 ± 4.3% of COUP-TFII-positive interneurons. Virtually, all reelin-positive interneurons were COUP-TFII-positive (98 ± 2%) and these included CCK-positive cells (29.7 ± 4.2% of reelin-positive cell and 23.0 ± 9.5% of CCK-positive interneurons). Reelin-positive cells were located most superficially; 85.3% ± 2.3% of them were in layers I and upper II (Fig. 4*B*, bins 1–2) sharply decreasing in abundance below layer II. The reelin- and COUP-TFII-positive cells represented less than half (40.1 ± 6.9%) of the total COUP-TFII population in bins 1–2 (Fig. 4*B*), most of the other cells were positive for CCK and/or calretinin. In layer I, some COUP-TFII-/reelin-positive interneurons were also positive for calretinin, and all these interneurons were CCK-negative. Thus, the expression of calretinin and CCK appears to be mutually exclusive in reelin-positive interneurons. In the supragranular layers, pyramidal cells did not express immunohistochemically detectable COUP-TFII (Figs 1, 2, and 3*B*).

Additional, widely expressed interneuron class-specific molecules include parvalbumin, calbindin, and somatostatin. These were tested in sections from all 3 patients in a quadruple immunoreaction, but none of the neurons immunopositive for these molecules (*n* = 110 for parvalbumin, *n* = 235 for calbindin, and *n* = 81 for somatostatin) contained immunohistochemically detectable level of COUP-TFII protein (Fig. 3*C*), even though the area evaluated contained 194 COUP-TFII-positive interneurons. Somatostatin was often colocalized with calbindin (Fig. 3*C*), and some of the parvalbumin-positive neurons also expressed calbindin immunoreactivity (not shown) as has been previously found in the human cortex (Gonzalez-Albo et al. 2001) as well as in the rodent cortex (Blatow et al. 2003).

In layers V and VI, many pyramidal cells visualized either by immunoreactivity for NPNFP or CCK were also positive for COUP-TFII (Fig. 3*E*). In general, the intensity of COUP-TFII immunoreactivity in pyramidal cells was weaker than in interneurons and highly variable down to an undetectable level, which made it difficult to estimate the fraction of immunopositive pyramidal cells.

### Electrophysiological Recording of COUP-TFII-Positive Interneurons In Vitro

The immunohistochemical characterization described above by itself does not allow the testing of how COUP-TFII-positive interneurons integrate into the synaptic network of the human cortex. Because the results show that COUP-TFII-positive interneurons may belong to several cell types, we sought to visualize individual cells to reveal their axonal and dendritic distributions and test their synaptic output. The latter has been shown to be a defining characteristic of distinct cell types in nonhuman animals (Szentagothai and Arbib 1974; Somogyi 1977; Thomson et al. 2002; Karube et al. 2004) as well as in man (Kisvarday et al. 1986). We have recorded visually identified neurons in vitro from layers II to upper III, visualized them by biocytin labeling, and tested them for COUP-TFII immunoreactivity. Seventeen of 62 tested interneurons were COUP-TFII-positive. The characterization of COUP-TFII-immunonegative neurons is beyond the scope of this study; here, we analyze only the COUP-TFII-positive interneurons. We tested them for the presence of molecules that were found to colocalize with COUP-TFII in the population survey presented above. The results are presented in Table 3. Reelin could not be tested in sections from tissue slices incubated in vitro, because the signal amplification technique produced very widespread pyramidal cell labeling with this antibody that could not be interpreted, although it produced selective interneuron labeling in fresh tissue sections.

**Table 3:** Immunoreaction tests and electrophysiological parameters of interneurons recorded in vitro in layers I–III

Inter neuron no.	Cell code	Patient code	Soma in layer	COUP-FTII	Immunoreaction		Recovered HRP reaction	*V*_m_ (mV)	*R*_in_ (MΩ)	Tau (ms)	Sag*	Action potential				Illustration
					Calretinin	CCK						Ampl. (mV)	Half width (ms)	Threshold (mV)	After hyperpolarization (mV)	
1	061101-1s	1	III	+	+	−	d	−71.1	437.3	6.1	+	76.6	0.84	−48.3	−18.6	
2	061109-1s	2	III	+	+	n.t.	d	−71.3	79.5	4.9	+	63.9	0.25	−38.6	−24.1	Figure 8
3	061101-3s	1	II	+	+	−	d	−72.2	277.7	8.1	+	75.4	0.58	−55.0	−9.3	
4	061101-1cs	1	II	+	+	−	−	−67.9	297.8	8.6	+	51.2	0.91	−31.5	−18.0	
5	061124-5cs	3	II	+	−	+	s, d, a	−68.8	194.6	6.4	+	53.4	0.83	−37.5	−14.3	Figure 7
6	070216-4cs	5	II	+	+	−	s, d	−75.1	302.0	8.8	−	74.5	0.46	−48.5	−15.5	
7	070216-5s	5	II	+	+	n.t.	s, d, a	−69.3	262.5	8.6	+	86.7	0.62	−61.1	−15.4	
8	070403-3cs	6	II	+	+	n.t.	s, d, a	−91.3	77.5	6.0	−	n.a.	n.a.	n.a.	n.a.	Figure 6
9	070403-1mg	6	II	+	+	n.t.	s, d, a	−74.0	194.0	5.7	+	91.0	0.43	−41.1	−13.5	Figure 7
10	070517-3kg	7	III	+	−	+	s, d, a	−71.3	244.1	9.5	+	63.6	0.74	−35.2	−17.9	
11	070517-2cs	7	II	+	−	+	s, d, a	−70.0	280.7	7.1	+	58.9	0.56	−34.2	−16.8	Figure 5*A*
12	070517-5cs	7	II	+	+	−	s, d, a	−72.9	321.7	9.0	−	68.7	0.35	−36.2	−26.9	Figure 7
13	070613-2sz	8	III	+	n.t	n.t.	s, d, ?	−78.2	294.7	11.2	+	64.1	0.42	−43.4	−24.9	
14	070613-1cs	8	II	+	−	n.t.	s, d	−67.0	271.7	10.1	+	67.5	0.48	−40.8	−18.9	
15	070613-1mg	8	II	+	−	n.t.	s, d, a	−74.5	176.8	8.3	+	64.2	0.51	−45.6	−16.9	
16	070608-4cs	9	I	+	−	−	s, d, a	−69.6	117.5	4.3	+	63.1	0.36	−39.8	−27.5	Figure 7
17	070824-3cs	10	II	+	+	n.t.	s, d, a	−76.5	183.9	4.9	+	79.7	0.44	−43.5	−20.8	Figure 5*B*
							Mean	−73.0	236.1	7.5		68.9	0.55	−42.6	−18.7	
							SD	5.6	93.1	2.0		11.1	0.19	7.8	5.0	

s: soma; d: dendrites; a: axon; n.t.: not tested, n.a.: not available.

*In response to hyperpolarizing current injection.

**Figure 5.**
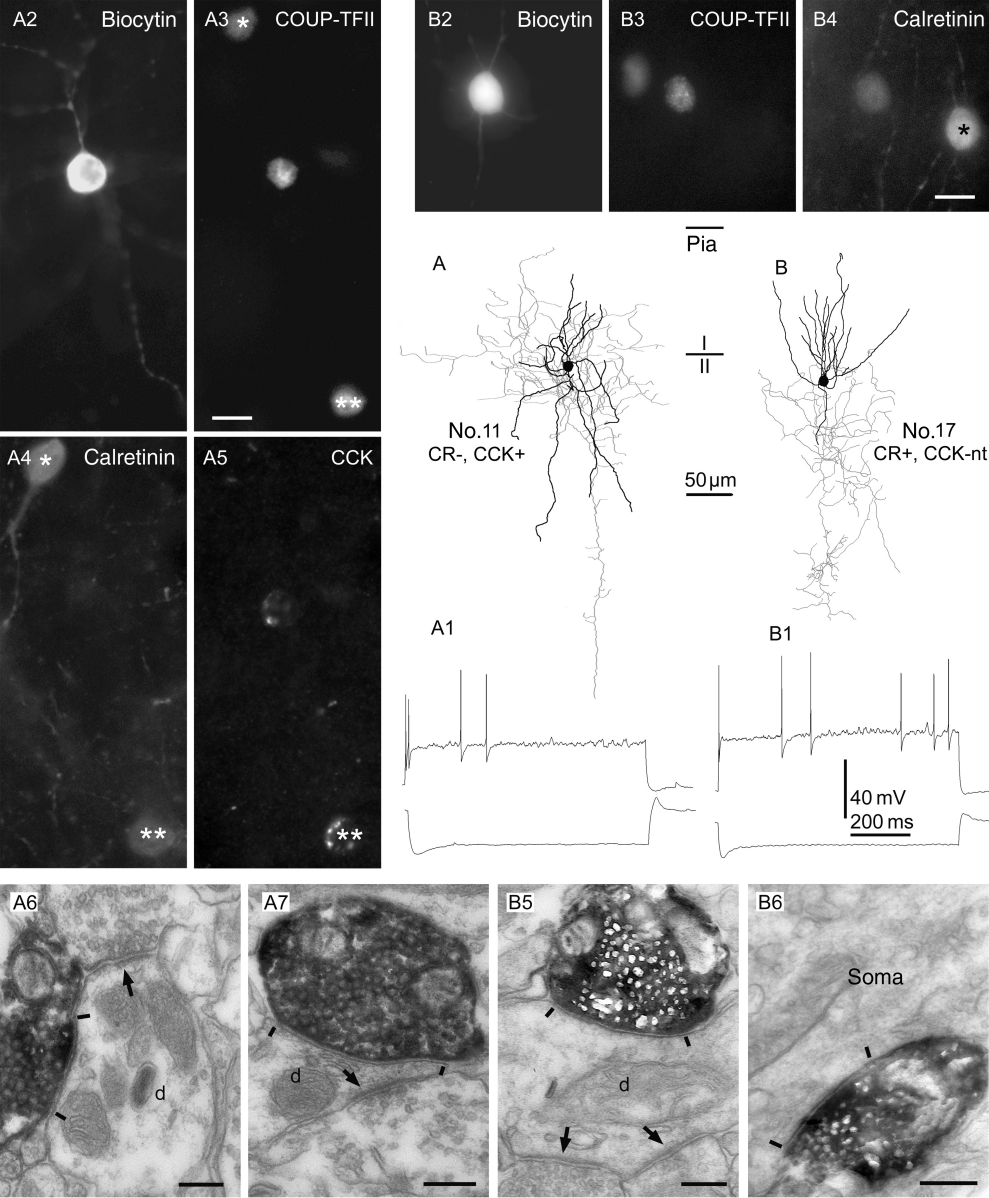
Interneurons immunopositive for COUP-TFII show irregular firing patterns in vitro and innervate mostly small dendrites*.* (*A* and *B*) Axonal (gray) and dendritic (black) distribution of 2 COUP-TFII-immunopositive cells in layer II. (*A1* and *B1*) Both cells responded with a robust sag to hyperpolarizing current steps (bottom traces), and fired irregular spikes to depolarizing current steps (top traces). (*A2–A5* and *B2–B4*) Cell A was immunopositive for CCK, but negative for calretinin, whereas cell B was positive for calretinin; it was not tested for CCK. Note 2 COUP-TFII-/calretinin-positive interneurons in *A*3 and *4* (asterisks), one of which was also positive for CCK (double asterisks). In *B3* and *4*, a rare calretinin-positive interneuron is seen that was immunonegative for COUP-TFII (asterisk). (*A6,7* and *B5,6*) Electron micrographs showing large, type II synaptic junctions (between bars) established by the cells shown in (*A*) and (*B*), respectively, with small dendritic shafts (d) and an interneuron soma. The innervated dendrites also receive type I synapses (arrows) from other boutons. Scale bars: *A* and *B*, 10 µm; *A6*,*7* and *B5*,*6*, 200 nm.

Sixteen COUP-TFII-positive interneurons were tested for calretinin and 10 (62.5%) were immunopositive (Figs 5*B2-4*,6 and 8*B*,*C*,*D*). Five of these were also tested for CCK immunoreactivity and they were all immunonegative. Ten COUP-TFII-immunoreactive interneurons were tested for CCK immunoreactivity and 3 (30%) were immunopositive (Fig. 5*A2-5*). All 3 were immunonegative for calretinin. One additional interneuron was immunonegative for both CCK and calretinin (Table 3).

**Figure 6.**
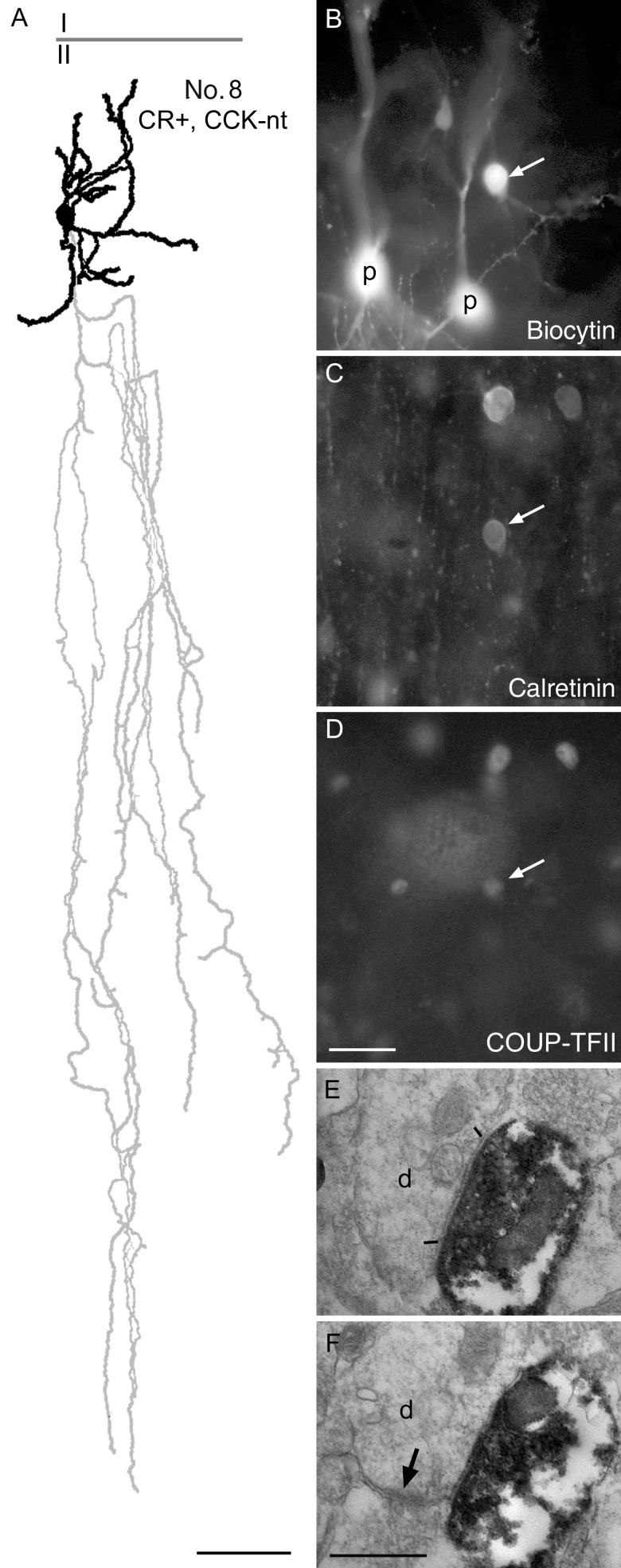
Demonstration of COUP-TFII immunoreactivity in a double bouquet cell. (*A*) Reconstruction of the dendritic (black) and axonal (gray) arborizations of the cell with the boundary between layers I and II indicated. Note the descending axonal bundle identifying the cell as a double bouquet cell. Most dendritic and axonal branches are truncated by the slice preparation. (*B*) The biocytin-labeled cell (arrow) next to 2 sequentially recorded pyramidal cells (P), which were tested for connectivity, but there was no detectable synaptic interaction between the interneuron and either of the pyramidal cells. (*C*) The double bouquet cells (arrow), but not the pyramidal cells, are immunopositive for calretinin. (*D*) The nucleus of the cell (arrow) is positive for COUP-TFII, as are the nuclei of 2 other calretinin-positive interneurons (top). (*E* and *F*) Electron micrographs of representative serial sections of a double bouquet cell bouton making a type II synaptic junction (between bars) with a small dendritic shaft that also receives another synapse (arrow) from a bouton of unknown origin. Scale bars: *A*, 50 µm; *B–D*, 25 µm; *E* and *F*, 0.5 µm.

**Figure 7.**
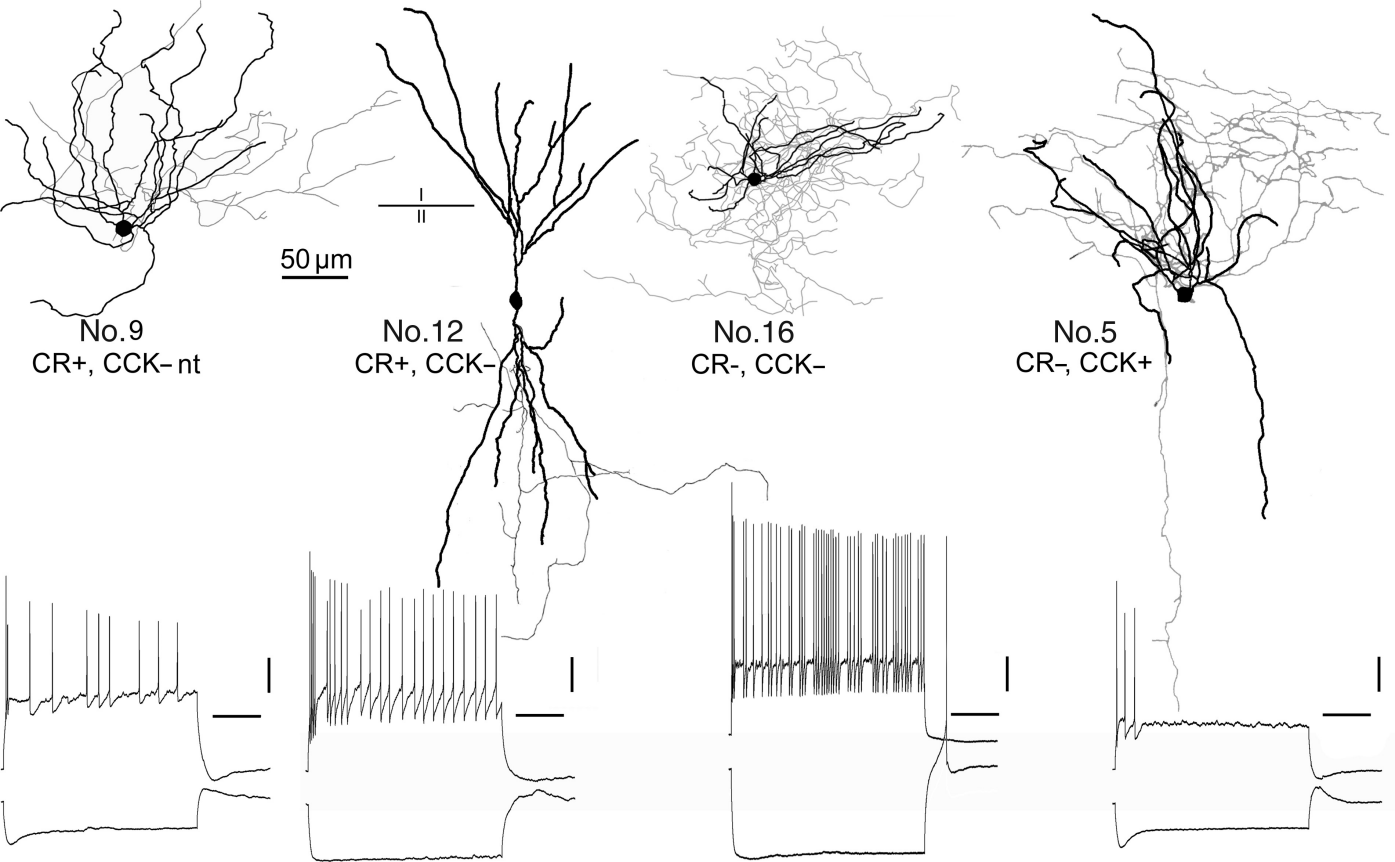
COUP-TFII-immunopositive interneurons in layer II, showing different dendritic (black) and axonal (gray) arborizations, and responses to depolarizing and hyperpolarizing current injections at resting membrane potential (lower row). The neurons showed highly variable firing patterns, spike frequency adaptation and spike amplitude accommodation, and sag and depolarizing hump in response to hyperpolarization. Cells were visualized by intracellular biocytin injection following whole-cell recording. All cells were tested for CR immunoreactivity; Nos 9 and 12 were immunopositive; the other 2 neurons were immunonegative. Cells 12, 16, and 5 were tested for CCK and only cell 5 was immunopositive. Scale bars: drawings, 50 µm; recordings, 20 mV, 200 ms.

After converting biocytin fluorescence to a horseradish peroxidase reaction product using DAB as a chromogen, 13 somata and 16 full or partial dendritic arborizations were recovered (Table 3). Some somata, dendrites, and axons were lost from those sections that had been treated with Sudan B to reduce autofluorescence, and some axons and dendrites were lost due to the slice preparation. The axons of 4 calretinin- and 3 CCK-positive cells could be evaluated. These 7 interneurons were reconstructed (Figs 5–7). One neuron was immunonegative for both calretinin and CCK, and its soma was located at the border of layers I and II (Fig. 7, No. 16). The dendritic tree was mainly horizontal and the dense axon innervated layers I and II equally. The large boutons made this cell distinguishable from neurogliaform cells frequent in layer I rodents (Hestrin and Armstrong 1996).

The 3 CCK-expressing cells resembled each other in that they had round somata, small compact dendritic trees either of a bushy (Fig. 7, No. 5) or multipolar (Fig. 5*A*) shape. Their axons were concentrated in layer II with less innervation of the adjoining layers I and III; the lack of descending axons did not seem to result from truncation during the slice preparation. The large round boutons did not form any conspicuous formations such as baskets or radial bundles.

**Figure 8.**
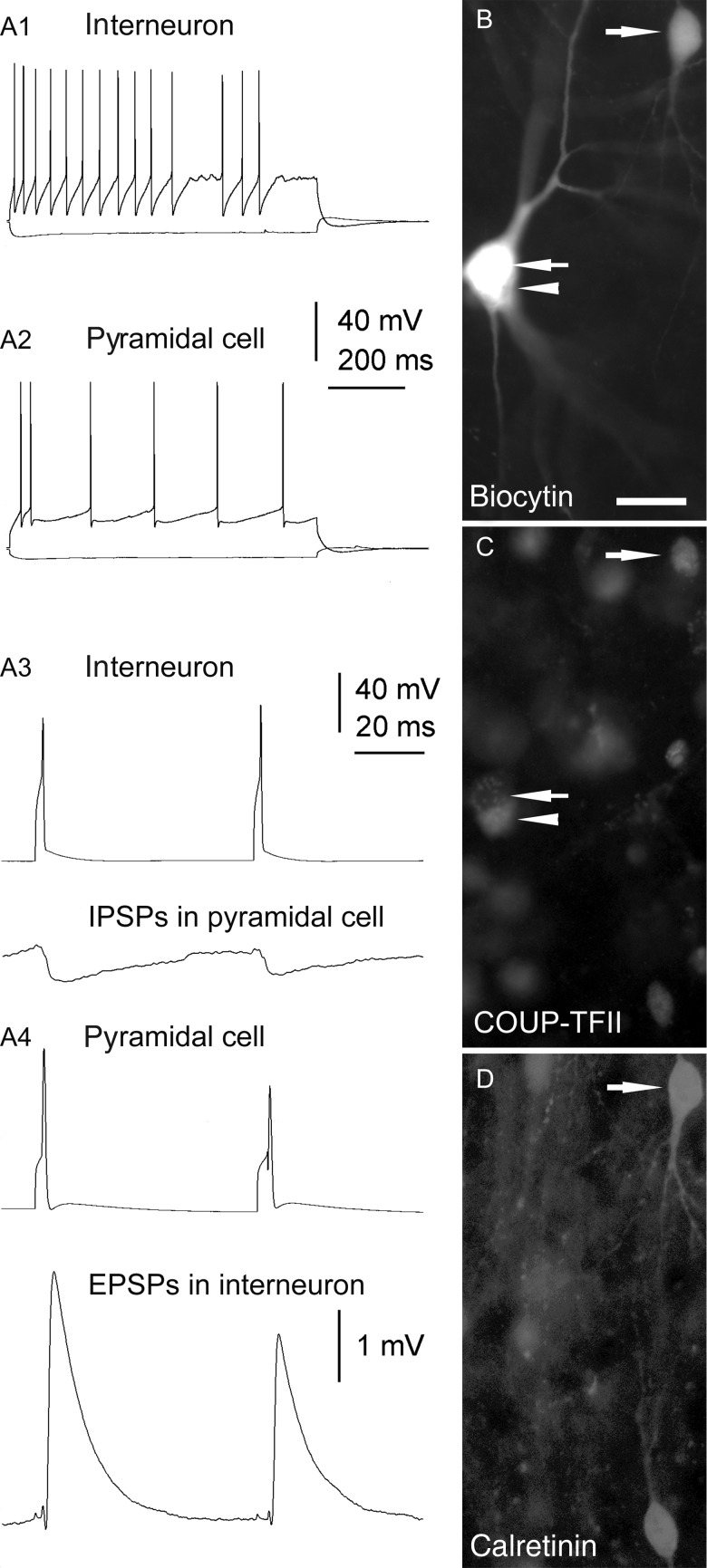
Reciprocal synaptic connections between a calretinin-/COUP-TFII-positive interneuron and a pyramidal cell in layer II. (*A1* and *A2*) AP patterns of the 2 neurons in response to depolarizing (top traces) or hyperpolarizing (bottom traces) current steps. (*A3* and *A4*) APs of the interneuron (*A3*, top trace) or the pyramidal cell (*A4*, top trace) elicit IPSPs (*A3*, bottom trace) or EPSPs (*A4*, bottom trace), respectively, in the postsynaptic cell. IPSPs and EPSPs are averages of 20 sweeps. (*B*) The biocytin filled interneuron (upper right corner) and pyramidal cell (arrow). (*C*) The nucleus of the interneuron, but not that of the pyramidal cell, is immunopositive for COUP-TFII. Arrowhead points to a COUP-TFII-positive interneuron in close apposition to the pyramidal cell. Note weak cytoplasmic autofluorescence in the pyramidal cell. (*D*) The interneuron as well as another COUP-TFII-positive cell (open arrow) are immunopositive for calretinin. Scale bar: 20 µm.

The calretinin-positive cells were more variable. The somata were mostly radially elongated fusiform-shaped (*n* = 6; Figs 7 and 8) or round to ovoid (*n* = 4; Figs 5*B* and 7, No. 9). The dendritic trees were mostly bitufted (*n* = 5; Figs 6, No. 8 and 7, No. 12) or bushy with mostly ascending dendrites (*n* = 3; Figs 5*B*, No. 17 and 7, No. 9). The axon of one calretinin-positive interneuron (Table 3, No. 9; Fig. 6) showed the typical descending axon bundle of double bouquet cells (Ramon y Cajal 1899; Jones 1975; Somogyi and Cowey 1981; del Rio and DeFelipe 1995). The other cells with sufficient axon for evaluation showed more locally arborizing loosely arranged axons in layers II and III without any particular grouping of the boutons.

The COUP-TFII-positive interneurons had a resting membrane potential of −73.0 ± 5.6 mV, membrane input resistance of 236.1 ± 93.1 MΩ, and a membrane time constant of 7.5 ± 2.0 ms (all *n* = 16). With 3 exceptions (Table 3), all interneurons responded with a robust sag to hyperpolarizing current steps (Figs 5*A1*,*B1* and 7). Many cells (*n* = 8) showed a depolarizing hump following a hyperpolarizing current injection step (Figs 6*A1*,*B1* and 7), and 4 cells responded with a rebound spike (Fig. 7). Parameters of individual APs were relatively heterogeneous in terms of AP amplitude (68.9 ± 11.1 mV), half width (0.55 ± 0.19 ms), threshold (−42.6 ± 7.8 mV), and afterhyperpolarization amplitude (18.7 ± 5.0 mV, relatively to AP threshold). At rheobasic current steps, 8 cells fired a single AP at the onset and remained silent during the rest of the 800-ms depolarization, and 7 cells fired continuously throughout the current pulse. When increasing the current amplitude, 10 cells fired continuously during the entire pulse, 7 cells showed spike frequency adaptation, and 3 cells had spike amplitude accommodation. After the onset of firing, some cells showed an irregular, stuttering firing pattern or remained silent.

Of the 10 cells with sufficient axons, 5 cells had axons in detergent-treated sections processed for immunocytochemistry; therefore, their synaptic targets could not be tested by electron microscopy. Electron microscopic analysis of synaptic targets was attempted on 5 cells and 3 had adequate tissue preservation for further analysis, and these (Cells 8, 11, and 17, Table 3) were studied quantitatively. To increase immunoreactivity, the fixative used in this study did not include glutaraldehyde that is used conventionally in most electron microscopic studies of the brain. We have found that the addition of picric acid to the paraformaldehyde fixative improved not only the immunoreactivity (Somogyi and Takagi 1982), but also the fine structural preservation of synaptic boutons. Interneurons 11 and 17 made relatively large type II synaptic junctions (Figs 5*A6*,*7* and *B5*,*6*), with a mean largest linear extent of 517 ± 122 nm (Fig. 5 cell A, *n* = 14) and 405 ± 94 nm (Fig. 5 cell B, *n* = 22). The large boutons (*n* = 14 tested) of the calretinin-positive cell (No. 17, Table 3), illustrated in Figure 5*B*, innervated mainly small dendritic shafts (72%), one dendritic spine, and one soma (Fig. 5*B6*) of an interneuron with 2 synapses; 4 (16%) postsynaptic targets could not be identified, as they were either small dendritic shafts or spines. The postsynaptic interneuron was identified on the basis of having a small amount of somatic cytoplasm, an invaginated nucleus, and no apical dendrite. The CCK-positive cell (No. 11, Table 3), illustrated in Figure 5*A*, only innervated small dendritic shafts (*n* = 25). Many of the dendritic shafts innervated by the boutons of these cells received 1 or 2 other (64% cell A and 50% cell B), mostly type I synapses (83%; Fig. 5*A6*,*A7*,*B5*) and rarely another type II synapse from boutons of unknown origin within the serial section that included the identified synapse. The putative double bouquet cell (No. 8, Table 3) also made synapses mainly with small dendritic shafts (Fig. 6*D*,*E*) 9 of which were identified; one small postsynaptic target was either a small dendrite or a spine. Of the dendritic shafts, 50% received mainly type I synapses from other boutons (Fig. 6*E*). Some of the dendritic shafts resembled those of interneurons described in nonhuman mammalian cortex. However, in the absence of adequate knowledge and due to currently undefined differences in the synaptic inputs to the dendritic shafts of pyramidal cells and interneurons in the human cortex, we could not establish the pyramidal cell or interneuron origin of these dendrites quantitatively.

Based on previous results from animals, we assumed that the COUP-TFII-positive interneurons were GABAergic, and sought to test this in paired recording of potential postsynaptic targets. Out of numerous paired recordings involving interneurons, in one case a reciprocal monosynaptic connection between a pyramidal and a COUP-TFII/calretinin-immunoreactive cell (Fig. 8) was found. In the reciprocal connection, APs of the interneuron evoked a relatively slow hyperpolarizing postsynaptic response in the pyramidal cell [mean first inhibitory postsynaptic potentials (IPSP) amplitude, 0.52 mV at – 47.2 mV membrane potential], and these showed a small paired pulse depression (IPSP2/IPSP1 = 0.8). The hyperpolarizing nature of the postsynaptic response is consistent with the action of GABA. Action potentials in the pyramidal cell evoked large depolarizing EPSPs [mean first excitatory postsynaptic potential (EPSP) amplitude, 3.4 mV at – 43.8 mV membrane potential], which showed paired pulse depression (EPSP2/EPSP1 = 0.74). Unfortunately, the cell pair could not be recovered for reconstruction due to the Sudan Black B treatment that preceded the immunoreaction and interfered with the peroxidase reaction using DAB as a chromogen.

## Discussion

Distinct types of cortical GABAergic interneuron occupy specific spatial and temporal positions in the cerebral cortex, orchestrating the information carrying activity of pyramidal cells. Not surprisingly, several neurological and psychiatric disorders involving the cerebral cortex are thought to be associated with abnormal ontogenetic developmental and/or maladaptive changes in interneuron activity (Rubenstein and Merzenich 2003; Marin 2012; Lewis 2014). Recognizing the identities of interneuronal cell types is a prerequisite for the explanation of circuitry in the cortex. Defining interneuron types and their roles in the cortical circuits in rodents has advanced considerably (DeFelipe et al. 2013), but information for the human cortex is rare (Kisvarday et al. 1986; del Rio and DeFelipe 1997a, 1997b; Molnar et al. 2008).

Throughout the central nervous system, the identification of cell lineage-specific nuclear transcription factors has facilitated the delineation of cell types (Hinoi et al. 2002; Marmigere and Ernfors 2007; Kessaris et al. 2014). In the cerebral cortex, clear differences have been established between pyramidal cells and GABAergic interneurons, most of the latter originating from the ganglionic eminences of the subpallium (Xu et al. 2004; Miyoshi et al. 2013; Kessaris et al. 2014; Marin and Muller 2014). Distinct transcription factors in the progenitor cell populations contribute to the migration and to the phenotypic maturation of interneurons. One of these, COUP-TFII contributes to the migration of interneurons, both in the rodent (Kanatani et al. 2008) and in the human cortex (Ma et al. 2013), and COUP-TFII is also strongly expressed in some populations of adult interneurons as also shown here. Neurons expressing a transcription factor in the adult are not necessarily the same cells that also expressed it when they were born. Here, we used the expression of COUP-TFII in combination with other molecules involved in cell signaling to delineate several interneuron populations in well-preserved surgical biopsies. By detecting up to 4 molecules in the same section, we have accounted for all COUP-TFII-expressing interneurons and report several novel molecular combinations. The main findings are: (1) COUP-TFII is present in specific populations of interneurons and pyramidal cells in the adult cortex; (2) most of the COUP-TFII-immunoreactive interneurons are calretinin- and/or reelin-positive cells that fire irregularly; (3) the co-expression patterns of calretinin, reelin, and CCK show specific combinations in adult human interneurons; (4) COUP-TFII-/calretinin-positive interneurons innervate dendrites, evoke IPSPs in pyramidal cells, and receive reciprocal EPSPs from them; (5) the widely recognized parvalbumin, somatostatin, and calbindin-expressing interneuron populations were immunonegative for COUP-TFII.

### Developmental Origin of COUP-TFII-Positive Interneurons

Using cell lineage-specific transcription factors, Ma et al. (2013) concluded that, in human and monkey cortex, the majority of GABAergic interneurons originate from the subpallium, in the MGE, LGE, and CGE, as shown by previous studies in rodents (Ma et al. 2012; Cai et al. 2013; Kessaris et al. 2014; Marin and Muller 2014). Other studies emphasized that in primates including humans, a large fraction of interneurons is generated in the ventricular and subventricular zones of the dorsal forebrain (Letinic et al. 2002; Petanjek et al. 2009; Jakovcevski et al. 2011). In particular, COUP-TFII is expressed in progenitor cells of both the human CGE and the ventricular/subventricular proliferative zones (Reinchisi et al. 2012). In mice, the transcription factors Sp8, Sox6, and COUP-TFII accounts for 90% of cortical interneuron (Ma et al. 2012), and about 30–40% of interneurons derive from the CGE (Miyoshi et al. 2013; Kessaris et al. 2014). The COUP-TFII-expressing interneurons in rodents are a distinct but diverse population (Kanatani et al. 2008; Vucurovic et al. 2010; Ma et al. 2012; Cai et al. 2013; Kessaris et al. 2014). In the full term developing human cortex, COUP-TFII-positive interneurons show the same laminar distribution (Ma et al. 2013) as in the adult cortex in our study. Furthermore, we have detected COUP-TFII immunoreactivity in a distinct set of deep layer pyramidal cells. The role of COUP-TFII in the adult cortex is not clear. During development, it is coexpressed with PROX1, a downstream transcription factor (Rubin and Kessaris 2013), present in the LGE/CGE and some preoptic area-derived interneurons.

### Molecular Expression Patterns in Cortical Interneurons Immunopositive for COUP-TFII

The final adult phenotypic manifestation of a cell type is due to a common ontogenetic origin and expression of signaling machinery, which arise through common repertoires of transcription factors. Interneurons expressing COUP-TFII have been shown to express calretinin, vasoactive intestinal polypeptide (VIP), somatostatin, and reelin in various mammals (Kanatani et al. 2008; Vucurovic et al. 2010; Reinchisi et al. 2012; Ma et al. 2013; Kessaris et al. 2014). Although useful for differentiating groups of neurons, none of these molecules delineate a single interneuron type on their own. Here, we have extended the analysis to novel combinations of molecular markers including CCK.

There is no agreement about what constitutes a cortical neuronal type (Thomson et al. 2002; Toledo-Rodriguez et al. 2005; Ascoli et al. 2008; Zeng et al. 2012; DeFelipe et al. 2013). A simple definition is that “Two individual neurons belong to the same cell type if they deliver the same neuroactive substances to the same range of postynaptic targets in the same temporal pattern in a brain state-specific manner” (Somogyi 2010). In practice, to obtain such multifaceted information for any cortical neuron is hard (Katona et al. 2014; Petersen 2014) and partial measures are used as surrogates to delineate populations of neurons. In most cases, the expression of a single molecule, or a small combination of molecules, such as calcium-binding proteins, or neuropeptides, or the presumed efferent synaptic sites such as “perisomatic” or “dendritic” are used to group, study, and genetically manipulate interneurons in an attempt to predict their function, as if they were a single-cell type with a unified role. However, in reality, each molecule is expressed in different combinations with others in cell types with distinct synaptic connections as reflected in the shapes of their axons (Ramon y Cajal 1899; Szentagothai and Arbib 1974; Thomson et al. 2002). Furthermore, the dendritic or axonal shapes alone without some molecular or temporal signature have not led to the unambiguous definition of interneuron types with one exception, the axo-axonic cell (Somogyi et al. 1982). Thus, basing conclusions on a single molecule or shape alone leaves significant uncertainty in interpretations (Cauli et al. 2014). For example, neither parvalbumin, nor somatostatin, is expressed by one interneuron type defined in terms of synaptic input/output connections (Somogyi 2010; Kubota 2014). The connections, in turn, show differences in function evident in the temporal dynamics of GABA release to often completely segregated subcellular domains of the postsynaptic pyramidal cells, for example, from parvalbumin-expressing axo-axonic and basket cells throughout the cortex. Nevertheless, at our current limited level of knowledge, grouping cells on one or a combination of expressed molecules leads to progress (Toledo-Rodriguez et al. 2005), as long as oversimplifications are avoided.

*Calretinin* is present in the largest population of COUP-TFII-expressing neurons in both rodents (Kanatani et al. 2008; Ma et al. 2012; Cauli et al. 2014; Kessaris et al. 2014) and human cortex (Reinchisi et al. 2012; Ma et al. 2013), as also shown here. In the human medial prefrontal cortex (Gabbott, Jays, et al. 1997) and temporal cortex (Gonzalez-Albo et al. 2001), calretinin-positive interneurons are particularly numerous in layers I–III, as also shown here, and some express VIP (Gabbott and Bacon 1997). In rodents, many, but not all calretinin-positive neurons express somatostatin (Xu et al. 2006), VIP (Rogers 1992; Kubota et al. 2011), and corticotropin-releasing factor (CRF, Kubota et al. 2011), neuropeptides that we did not test here. Unlike in the rodent (Cauli et al. 2014), a significant proportion of human calretinin-/COUP-TFII-positive cells is also positive for CCK.

*Reelin* is a key signaling protein secreted during development by Cajal–Retzius cells (Ogawa et al. 1995; Hammond et al. 2006) and its expression is also retained in distinct GABAergic neurons in the adult cortex (Alcantara et al. 1998; Fuentealba et al. 2010). Reelin-positive cells constituted approximately 20% of the total COUP-TFII-positive population. The coexpression of a high level of alpha-actinin2 and CCK or CR in layer I neurons in the rat (Uematsu et al. 2008), similar to that for the human shown here for CCK and CR, points to neurogliaform cells as a major population. Layer I contains a large diversity of interneurons both in terms of the coexpression of molecules (Kubota et al. 2011) and cell types defined by other features (see below).

*Cholecystokinin-*expressing small interneurons were approximately 20% of all COUP-TFII-positive cells and were identified here with antibody to pro-CCK (Morino et al. 1994) and showed a diverse combination of calretinin and reelin expression. Interneurons expressing CCK are not well characterized in any species, and CCK is also expressed by some pyramidal cells (Morino et al. 1994) as also shown here in the human. In rodents, VIP and/or CRF is coexpressed in some, but not all CCK-positive interneurons (Kubota et al. 2011), which remain to be tested in the human cortex.

*Somatostatin* immunoreactivity was not present in the COUP-positive neurons tested here, but a previous report described it in interneurons in the deep cortical layers of the mouse and suggested that they originate from the dLGE (Cai et al. 2013). The type of somatostatin and COUP-TFII coexpressing neuron is not known. Some but not all deep layer somatostatin neurons send axons to layer I and are named Martinotti cells. However, others close by express somatostatin, neuropeptide tyrosine, and nitric oxide synthase, and project widely throughout all layers and cortical areas (Tomioka et al. 2005).

In terms of their intrinsic electrophysiologial parameters, the COUP-TFII-expressing cells are quite heterogeneous. They vary in AP amplitude, half width, and accommodation. The only common feature among all recorded COUP-TFII-positive interneurons with respect to their electrophysiological properties is their response with a robust sag to hyperpolarizing currents steps. This, however, seems to be a general feature of most human cortical interneurons (Molnar et al. 2008).

### Synaptic Specializations of Cortical Interneuron Types

How do the cortical interneuron populations having partially or completely different neurochemical composition map onto cell types defined by synaptic connectivity remains a challenge in any species (Toledo-Rodriguez et al. 2004; Zeng et al. 2012; DeFelipe et al. 2013; Pfeffer et al. 2013), and key missing information in the human cortex. The COUP-TFII-expressing cells characterized here in terms of molecular expression profiles, firing patterns in vitro, and axonal and dendritic arborizations (shape) have probably been seen in previous Golgi, immunohistochemical, and in vitro labeling studies. However, human cortical cell types remain poorly defined, and even in the rodent neocortex delineating distinct functional cell types (Kubota 2014) in terms of their synaptic connections is difficult. Below, we will try to draw together briefly the information that may delineate some interneuron types in the human cortex in light of the information gleaned from other animals.

*Axo-axonic cells* exclusively innervate the axon initial segment of pyramidal cells (Somogyi 1977), originating from the MGE in rodents (Inan and Anderson 2014) and often expressing parvalbumin. They have been documented in the human cortex (Kisvarday et al. 1986; Marin-Padilla 1987; Szabadics et al. 2006; Molnar et al. 2008). The lack of COUP-TFII immunoreactivity in parvalbumin-positive neurons suggests that axo-axonic cells are distinct from all COUP-TFII-expressing interneurons. *Basket cells* are defined as making >20% of their output synapses on somata of other neurons (Somogyi et al. 1983) and are highly diverse. They include parvalbumin (Blumcke et al. 1990; Kawaguchi and Kubota 1998) or CCK and VIP (Freund et al. 1986; Meyer and Wahle 1988; Kawaguchi and Kubota 1996; Kawaguchi and Kubota 1998)-expressing nonoverlapping basket cell populations. But not all parvalbumin- or CCK- or VIP-expressing interneurons are basket cells (Pfeffer et al. 2013). The presence of parvalbumin-expressing basket cells in the human cortex is clear from the presence of rich perisomatic terminal baskets (Blumcke et al. 1990), but as all parvalbumin-positive cell bodies were negative for COUP-TFII; this population of interneurons probably does not express COUP-TFII. We are unaware of information about the presence of CCK-expressing basket cells in the human cortex. The COUP-TFII-expressing CCK-immunopositive cells may have included basket cells, but the axons were too partially visualized to establish this, and the 2 CCK/COUP-TFII-positive small interneurons were not seen to terminate on somata by light microscopy. On the other hand, in neocortex of the rat (Gabbott, Dickie, et al. 1997) and the human (del Rio and DeFelipe 1997b; Gabbott, Jays, et al. 1997) as well as in the cat primary visual cortex (Meskenaite 1997), some pyramidal cell bodies are surrounded by calretinin-positive terminals. showing that some calretinin-positive neurons are basket cells. These basket cells may correspond to the significant population of calretinin-/CCK-/COUP-TFII-expressing neurons that we have identified here.

*Neurogliaform cells* are recognized from the extremely dense axonal arborization within and around the restricted dendritic arbor (Ramon y Cajal 1899; Szentagothai 1973; Olah et al. 2007; Kubota 2014). They co-express reelin and COUP-TFII in the rat hippocampus (Fuentealba et al. 2010), thus it is possible that many of the reelin-/COUP-TFII-positive neurons in the human cortex (Ma et al. 2013), and also found here particularly in layer I, are neurogliaform cells. In addition to their synaptic innervation of dendrites, they are thought to release GABA non-synaptically (Olah et al. 2007), and are a major source of slow GABA_A_ receptor-mediated postsynaptic responses (Tamas et al. 2003; Szabadics et al. 2007) and GABA_B_ receptor-mediated pre- and postsynaptic inhibition (Tamas et al. 2003; Craig and McBain 2014). They were shown to be GABA-immunopositive (Kisvarday et al. 1990) and evoke IPSPs (Olah et al. 2007) in the adult human cortex. Neurogliaform cells are most numerous in and close to layer I in rodents (Olah et al. 2007; Uematsu et al. 2008; Vucurovic et al. 2010). The densely localized axon of COUP-TFII-positive cell No. 16 and the short compact dendritic tree resemble those of neurogliaform cells in rodents (Olah et al. 2007) and human (Kisvarday et al. 1990). In rodents, neurogliaform cells show heterogeneous molecular expression profiles and may originate from both the MGE and the CGE (Tricoire et al. 2010), but the majority express high level of alpha-actinin2 (Uematsu et al. 2008). The coexpression of alpha-actinin2 and CCK or CR in layer I neurons in the rat (Uematsu et al. 2008) is similar to the pattern in COUP-TFII-positive neurons in the human described here. In addition to the reelin and COUP-TFII expression shown here, the expression of mRNA for insulin was also found in neurogliaform cells of the rat (Molnar et al. 2014). If found also in the human, it would point to neurogliaform cells as key players in normal or abnormal brain metabolism.

*Cajal–Retzius cells* occupy a subpial position in the developing cortical plate, are immunopositive for calretinin (Marin-Padilla 1998), secret reelin (Frotscher 1997), and express COUP-TFII (Tripodi et al. 2004). Most Cajal–Retzius cells degenerate and the number of reelin-expressing cells decreases dramatically during postnatal life (Del Rio et al. 1996; Radnikow et al. 2002). The GABAergic interneurons co-expressing combinations of calretinin, reelin, and COUP-TFII in the adult layer I, and likely secreting reelin (Alcantara et al. 1998; Martinez-Cerdeno and Clasca 2002), do not derive from Cajal–Retzius cells (Pesold et al. 1998), because the latter are glutamatergic neurons (Quattrocolo and Maccaferri 2014). These GABAergic neurons described here are likely to be newly arrived interneurons expressing some of the same molecules as do Cajal–Retzius cells. All reelin-immunopositive cells in layers I–III were COUP-TFII-positive in our analysis. In the rat barrel cortex, parvalbumin- and somatostatin-expressing interneurons are also reelin-immunopositive, which may indicate a species difference (Pohlkamp et al. 2014), as these neurons were not COUP-TFII-positive in our study.

*Double bouquet cells* are a heterogeneous population of neurons that innervate dendrites; the name is used for different and often poorly defined cell types in different studies, due to the lack of information about molecular characteristics and synaptic targets of individual neurons. Previously, we restricted the name to neurons with the soma in layers II/III and narrow columnar axons descending through all layers (Ramon y Cajal 1899, Figs 8 and 11; Somogyi and Cowey 1981; Somogyi and Cowey 1984). We have tentatively included the COUP-positive neuron No. 8 under this name based on the partial descending axonal bundle. They have been studied in nonhuman primates and human (Somogyi and Cowey 1981, 1984; Ballesteros-Yanez et al. 2005) and in the cat (Meyer 1983; Tamas et al. 1997), and are seen as forming the columnar axon bundles that make “horse tail formations.” Such 50–100 μm wide dense inhibitory axon bundles have featured prominently in schemes of so-called minicolumns. Recently, the name “double bouquet cell” has been applied to interneurons in rodents, but many of the cells so named may not be homologous to those in primates and humans. The expression of calretinin, calbindin, tachykinin, and CCK have been described in “double bouquet” cells (Freund et al. 1986; DeFelipe et al. 1989; 1990; del Rio and DeFelipe 1995; DeFelipe 1997; Ballesteros-Yanez et al. 2005), but the cells expressing these molecules may represent several different cell types. Uncertainty is due to the use of immunohistochemistry alone, which rarely allows the tracing of axons to immunopositive cell bodies and likely results in *en mass* labeling of many neuronal types. The expression of calbindin in double bouquet cells is particularly difficult to evaluate without single-neuron labeling, as layer II–III pyramidal cells also express calbindin and their descending axon bundles are entwined with the “horse tail” formations of “double bouquet” cells (del Rio and DeFelipe 1995). Based on the descriptions of synaptic connectivity, it is likely that “double bouquet” cells include at least 2 distinct cell types: (1) A type of interneuron that mainly innervates dendritic spines and also shafts of pyramidal cells (Somogyi and Cowey 1981; DeFelipe et al. 1989; DeFelipe et al. 1990; Tamas et al. 1997) with unknown molecular identity. (2) A distinct type of interneuron that targets other interneurons preferentially (Somogyi and Cowey 1981) and expresses calretinin (Meskenaite 1997). The calretinin and COUP-TFII co-expressing putative double bouquet cells described here (No. 8) could belong to the interneuron targeting type. The electron microscopic sample of postsynaptic targets of this cell is consistent with this proposal, as the detected dendritic shafts could belong to other interneurons. The other calretinin-positive neuron with a “double bouquet dendritic tree” (No. 12) as the term was originally used by Ramon y Cajal (1911) had little of its axon in the slice and could be of a similar interneuron type.

*Interneuron target-specific GABAergic interneurons* (*IS cells*) were discovered in the hippocampus (Freund and Buzsaki 1996). Some calretinin- and/or VIP-expressing interneurons preferentially target other interneurons in the neocortex of rodents [Dalezios et al. 2002; Caputi et al. 2009; Pfeffer et al. 2013; Pi et al. 2013; see Cauli et al. (2014)] and some calretinin-positive interneurons densely innervate other interneurons in the cat (Meskenaite 1997) and human (del Rio and DeFelipe 1997b). A recent elegant study named interneurons as “single bouquet cells” in layer I in the rat cortex without neurochemical characterization, but showed that they mainly inhibited other interneurons (Jiang et al. 2013). It is possible that these cells are the equivalent of interneuron innervating double bouquet axonal cells of layers II–III, but because the cell body is in layer I the ascending axon is no longer present.

One of the COUP-TFII/calretinin-positive cells identified here produced a slow monosynaptic IPSP in a pyramidal cell, and thus was not an exclusively interneuron-specific cell; the degree of selectivity of IS cells is not known for the human cortex. In the mouse cortex (Caputi et al. 2009), both VIP-expressing and VIP-negative calretinin-positive interneurons innervated both pyramidal cells and interneurons, and some VIP-positive interneurons preferentially, but not exclusively, innnervated somatostatin-expressing interneurons (Pfeffer et al. 2013).

### Synaptic Targets and Actions of COUP-TFII-Positive GABAergic Interneurons

The number and postsynaptic target element distribution of GABAergic terminals in the human cortex is not known. The synaptic target structures of the recorded COUP-TFII-expressing neurons show some similarities, with most boutons synaptically innervating dendritic shafts. In the macaque monkey primary visual cortex, dendritic shafts are the main synaptic targets of GABAergic terminals. Of the 76 million GABAergic synapses per mm^3^, 61% are on dendritic shafts, 12% on somata, and 27% on dendritic spines (Beaulieu et al. 1992). They comprise 17% of the 446 million synapses per mm^3^. The COUP-TFII-expressing neurons are a major contributor to dendritic shaft GABAergic innervation and a contributor to dendritic inhibition. We are unable to differentiate between dendritic shafts originating from pyramidal cells and interneurons, but the numerous type I synapses on some of the shafts innervated by the COUP-TFII-positive cells indicate that a proportion belongs to interneurons. The 2 CCK/COUP-TFII-positive cells recorded here had partial axons in the slice, but both of them innervated layer I abundantly, and they did not appear to provide pyramidal cell somatic innervation, which was confirmed by electron microscopy; hence they are not basket cells. One of the recorded COUP-TFII-/calretinin-positive interneurons innervated pyramidal cells, but this cell pair could not be investigated by electron microscopy because of the deleterious effect on the autofluorescence suppression solution that we used.

In conclusion, the exploration of COUP-TFII-expressing neurons in the human association cortex in terms of their molecular expression profiles and synaptic relationships have revealed a diverse GABAergic interneuron population, mainly innervating dendrites. Pyramidal cell dendrites carry out computations based on electrogenic supralinear integration of synaptic inputs, and dendritic inhibition can have a non-linear effect through the inhibition of dendritic spikes in a network and brain state-dependent manner (Gentet et al. 2012; Lovett-Barron and Losonczy 2014). Further work testing the effects of dendrite innervating interneurons in multiple recordings of synaptically connected cells in vitro could lead to a definition of these human cortical cell types.

## Funding

Funding to pay the Open Access publication charges for this article was provided by the Medical Research Council, UK.

## References

[BHV045C1] Alcantara S Ruiz M D'Arcangelo G Ezan F de Lecea L Curran T Sotelo C Soriano E 1998 Regional and cellular patterns of reelin mRNA expression in the forebrain of the developing and adult mouse. J Neurosci. 18:7779–7799. 9742148 10.1523/JNEUROSCI.18-19-07779.1998 PMC6792998

[BHV045C2] Alfano C Magrinelli E Harb K Studer M 2014 The nuclear receptors COUP-TF: a long-lasting experience in forebrain assembly. Cell Mol Life Sci. 71:43–62. 23525662 10.1007/s00018-013-1320-6 PMC11114017

[BHV045C3] Alkondon M Pereira EF Eisenberg HM Albuquerque EX 2000 Nicotinic receptor activation in human cerebral cortical interneurons: a mechanism for inhibition and disinhibition of neuronal networks. J Neurosci. 20:66–75. 10627582 10.1523/JNEUROSCI.20-01-00066.2000 PMC6774099

[BHV045C4] Ascoli GA Alonso-Nanclares L Anderson SA Barrionuevo G Benavides-Piccone R Burkhalter A Buzsaki G Cauli B DeFelipe J Fairén A 2008 Petilla terminology: nomenclature of features of GABAergic interneurons of the cerebral cortex. Nat Rev Neurosci. 9:557–566. 18568015 10.1038/nrn2402 PMC2868386

[BHV045C5] Ballesteros-Yanez I Munoz A Contreras J Gonzalez J Rodriguez-Veiga E DeFelipe J 2005 Double bouquet cell in the human cerebral cortex and a comparison with other mammals. J Comp Neurol. 486:344–360. 15846784 10.1002/cne.20533

[BHV045C6] Beaulieu C Kisvarday Z Somogyi P Cynader M Cowey A 1992 Quantitative distribution of GABA-immunopositive and -immunonegative neurons and synapses in the monkey striate cortex (area 17). Cereb Cortex. 2:295–309. 1330121 10.1093/cercor/2.4.295

[BHV045C7] Blatow M Rozov A Katona I Hormuzdi SG Meyer AH Whittington MA Caputi A Monyer H 2003 A novel network of multipolar bursting interneurons generates theta frequency oscillations in neocortex. Neuron. 38:805–817. 12797964 10.1016/s0896-6273(03)00300-3

[BHV045C8] Blumcke I Hof PR Morrison JH Celio MR 1990 Distribution of parvalbumin immunoreactivity in the visual cortex of old world monkeys and humans. J Comp Neurol. 301:417–432. 2262599 10.1002/cne.903010307

[BHV045C9] Buhl EH Tamas G Szilagyi T Stricker C Paulsen O Somogyi P 1997 Effect, number and location of synapses made by single pyramidal cells onto aspiny interneurones of cat visual cortex. J Physiol (Lond). 500:689–713. 9161986 10.1113/jphysiol.1997.sp022053 PMC1159419

[BHV045C10] Cai Y Zhang Q Wang C Zhang Y Ma T Zhou X Tian M Rubenstein JL Yang Z 2013 Nuclear receptor COUP-TFII-expressing neocortical interneurons are derived from the medial and lateral/caudal ganglionic eminence and define specific subsets of mature interneurons. J Comp Neurol. 521:479–497. 22791192 10.1002/cne.23186

[BHV045C11] Caputi A Rozov A Blatow M Monyer H 2009 Two calretinin-positive GABAergic cell types in layer 2/3 of the mouse neocortex provide different forms of inhibition. Cereb Cortex. 19:1345–1359. 18842664 10.1093/cercor/bhn175

[BHV045C12] Cauli B Porter JT Tsuzuki K Lambolez B Rossier J Quenet B Audinat E 2000 Classification of fusiform neocortical interneurons based on unsupervised clustering. Proc Natl Acad Sci USA. 97:6144–6149. 10823957 10.1073/pnas.97.11.6144 PMC18572

[BHV045C13] Cauli B Zhou X Tricoire L Toussay X Staiger JF 2014 Revisiting enigmatic cortical calretinin-expressing interneurons. Front Neuroanat. 8:52. 25009470 10.3389/fnana.2014.00052 PMC4067953

[BHV045C14] Cobos I Calcagnotto ME Vilaythong AJ Thwin MT Noebels JL Baraban SC Rubenstein JL 2005 Mice lacking Dlx1 show subtype-specific loss of interneurons, reduced inhibition and epilepsy. Nat Neurosci. 8:1059–1068. 16007083 10.1038/nn1499

[BHV045C15] Cobos I Long JE Thwin MT Rubenstein JL 2006 Cellular patterns of transcription factor expression in developing cortical interneurons. Cereb Cortex. 16:I82–I88. 16766712 10.1093/cercor/bhk003

[BHV045C16] Craig MT McBain CJ 2014 The emerging role of GABA_B_ receptors as regulators of network dynamics: fast actions from a “slow” receptor? Curr Opin Neurobiol. 26:15–21. 24650499 10.1016/j.conb.2013.10.002 PMC4024344

[BHV045C17] Dalezios Y Lujan R Shigemoto R Roberts JDB Somogyi P 2002 Enrichment of mGluR7a in the presynaptic active zones of GABAergic and non-GABAergic terminals on interneurons in the rat somatosensory cortex. Cereb Cortex. 12:961–974. 12183395 10.1093/cercor/12.9.961

[BHV045C122] de Bergeyck V Naerhuyzen B Goffinet AM Lambert de Rouvroit C 1998 A panel of monoclonal antibodies against reelin, the extracellular matrix protein defective in reeler mutant mice. J Neurosci Methods. 82:17–24. 10223511 10.1016/s0165-0270(98)00024-7

[BHV045C18] DeFelipe J 1997 Types of neurons, synaptic connections and chemical characteristics of cells immunoreactive for calbindin-D28K, parvalbumin and calretinin in the neocortex. J Chem Neuroanat. 14:1–19. 9498163 10.1016/s0891-0618(97)10013-8

[BHV045C19] DeFelipe J Hendry SHC Hashikawa T Molinari M Jones EG 1990 A microcolumnar structure of monkey cerebral cortex revealed by immunocytochemical studies of double bouquet cell axons. Neuroscience. 37:655–673. 1701039 10.1016/0306-4522(90)90097-n

[BHV045C20] DeFelipe J Hendry SHC Jones EG 1989 Synapses of double bouquet cells in monkey cerebral cortex visualized by calbindin immunoreactivity. Brain Res. 503:49–54. 2611658 10.1016/0006-8993(89)91702-2

[BHV045C21] DeFelipe J Lopez-Cruz PL Benavides-Piccione R Bielza C Larranaga P Anderson S Burkhalter A Cauli B Fairen A Feldmeyer D 2013 New insights into the classification and nomenclature of cortical GABAergic interneurons. Nat Rev Neurosci. 14:202–216. 23385869 10.1038/nrn3444 PMC3619199

[BHV045C22] del Rio MR DeFelipe J 1997a Double bouquet cell axons in the human temporal neocortex: relationship to bundles of myelinated axons and colocalization of calretinin and calbindin D-28k immunoreactivities. J Chem Neuroanat. 13:243–251. 9412906 10.1016/s0891-0618(97)00050-1

[BHV045C23] del Rio MR DeFelipe J 1995 A light and electron microscopic study of calbindin D-28k immunoreactive double bouquet cells in the human temporal cortex. Brain Res. 690:133–140. 7496800 10.1016/0006-8993(95)00641-3

[BHV045C24] del Rio MR DeFelipe J 1997b Synaptic connections of calretinin-immunoreactive neurons in the human neocortex. J Neurosci. 17:5143–5154. 9185552 10.1523/JNEUROSCI.17-13-05143.1997 PMC6573303

[BHV045C25] Del Rio JA Heimrich B Super H Borrell V Frotscher M Soriano E 1996 Differential survival of Cajal-Retzius cells in organotypic cultures of hippocampus and neocortex. J Neurosci. 16:6896–6907. 8824328 10.1523/JNEUROSCI.16-21-06896.1996 PMC6579265

[BHV045C26] Erbel-Sieler C Dudley C Zhou Y Wu X Estill SJ Han T Diaz-Arrastia R Brunskill EW Potter SS McKnight SL 2004 Behavioral and regulatory abnormalities in mice deficient in the NPAS1 and NPAS3 transcription factors. Proc Natl Acad Sci USA. 101:13648–13653. 15347806 10.1073/pnas.0405310101 PMC518807

[BHV045C27] Flames N Marin O 2005 Developmental mechanisms underlying the generation of cortical interneuron diversity. Neuron. 46:377–381. 15882635 10.1016/j.neuron.2005.04.020

[BHV045C28] Freund TF Buzsaki G 1996 Interneurons of the hippocampus. Hippocampus. 6:347–470. 8915675 10.1002/(SICI)1098-1063(1996)6:4<347::AID-HIPO1>3.0.CO;2-I

[BHV045C29] Freund TF Magloczky Z Soltesz I Somogyi P 1986 Synaptic connections, axonal and dendritic patterns of neurons immunoreactive for cholecystokinin in the visual cortex of the cat. Neuroscience. 19:1133–1159. 3029625 10.1016/0306-4522(86)90129-6

[BHV045C30] Frotscher M 1997 Dual role of Cajal-Retzius cells and reelin in cortical development. Cell Tissue Res. 290:315–322. 9321693 10.1007/s004410050936

[BHV045C31] Fuentealba P Klausberger T Karayannis T Suen WY Huck J Tomioka R Rockland K Capogna M Studer M Morales M 2010 Expression of COUP-TFII nuclear receptor in restricted GABAergic neuronal populations in the adult rat hippocampus. J Neurosci. 30:1595–1609. 20130170 10.1523/JNEUROSCI.4199-09.2010 PMC4487820

[BHV045C32] Gabbott PL Bacon SJ 1997 Vasoactive intestinal polypeptide containing neurones in monkey medial prefrontal cortex (mPFC): colocalisation with calretinin. Brain Res. 744:179–184. 9030431 10.1016/s0006-8993(96)01232-2

[BHV045C33] Gabbott PLA Dickie BGM Vaid RR Headlam AJN Bacon SJ 1997 Local-circuit neurones in the medial prefrontal cortex (areas 25, 32, and 24b) in the rat: morphology and quantitative distribution. J Comp Neurol. 377:465–499. 9007187 10.1002/(sici)1096-9861(19970127)377:4<465::aid-cne1>3.0.co;2-0

[BHV045C34] Gabbott PLA Jays PRL Bacon SJ 1997 Calretinin neurons in human medial prefrontal cortex (areas 24a,b,c, 32′, and 25). J Comp Neurol. 381:389–410. 9136798 10.1002/(sici)1096-9861(19970519)381:4<389::aid-cne1>3.0.co;2-z

[BHV045C35] Gentet LJ Kremer Y Taniguchi H Huang ZJ Staiger JF Petersen CC 2012 Unique functional properties of somatostatin-expressing GABAergic neurons in mouse barrel cortex. Nat Neurosci. 15:607–612. 22366760 10.1038/nn.3051

[BHV045C36] Gonzalez-Albo MC Elston GN DeFelipe J 2001 The human temporal cortex: characterization of neurons expressing nitric oxide synthase, neuropeptides and calcium-binding proteins, and their glutamate receptor subunit profiles. Cereb Cortex. 11:1170–1181. 11709488 10.1093/cercor/11.12.1170

[BHV045C37] Hammond V So E Gunnersen J Valcanis H Kalloniatis M Tan SS 2006 Layer positioning of late-born cortical interneurons is dependent on Reelin but not p35 signaling. J Neurosci. 26:1646–1655. 16452688 10.1523/JNEUROSCI.3651-05.2006 PMC6675480

[BHV045C38] Hestrin S Armstrong WE 1996 Morphology and physiology of cortical neurons in layer I. J Neurosci. 16:5290–5300. 8757242 10.1523/JNEUROSCI.16-17-05290.1996 PMC6578880

[BHV045C39] Hinoi E Balcar VJ Kuramoto N Nakamichi N Yoneda Y 2002 Nuclear transcription factors in the hippocampus. Prog Neurobiol. 68:145–165. 12450491 10.1016/s0301-0082(02)00078-3

[BHV045C40] Inan M Anderson SA 2014 The chandelier cell, form and function. Curr Opin Neurobiol. 26:142–148. 24556285 10.1016/j.conb.2014.01.009 PMC4024324

[BHV045C41] Jakovcevski I Mayer N Zecevic N 2011 Multiple origins of human neocortical interneurons are supported by distinct expression of transcription factors. Cereb Cortex. 21:1771–1782. 21139075 10.1093/cercor/bhq245 PMC3138511

[BHV045C42] Jiang X Wang G Lee AJ Stornetta RL Zhu JJ 2013 The organization of two new cortical interneuronal circuits. Nat Neurosci. 16:210–218. 23313910 10.1038/nn.3305 PMC3589105

[BHV045C43] Jones EG 1975 Varieties and distribution of non-pyramidal cells in the somatic sensory cortex of the squirrel monkey. J Comp Neurol. 160:205–268. 803518 10.1002/cne.901600204

[BHV045C44] Kanatani S Yozu M Tabata H Nakajima K 2008 COUP-TFII is preferentially expressed in the caudal ganglionic eminence and is involved in the caudal migratory stream. J Neurosci. 28:13582–13591. 19074032 10.1523/JNEUROSCI.2132-08.2008 PMC6671763

[BHV045C45] Karayannis T Au E Patel C Kruglikov I Markx S Delorme R Heron D Salomon D Glessner J Restituito S 2014 Cntnap4 differentially contributes to GABAergic and dopaminergic synaptic transmission. Nature. 511:236–240. 24870235 10.1038/nature13248 PMC4281262

[BHV045C46] Karube F Kubota Y Kawaguchi Y 2004 Axon branching and synaptic bouton phenotypes in GABAergic nonpyramidal cell subtypes. J Neurosci. 24:2853–2865. 15044524 10.1523/JNEUROSCI.4814-03.2004 PMC6729850

[BHV045C47] Katona L Lapray D Viney TJ Oulhaj A Borhegyi Z Micklem BR Klausberger T Somogyi P 2014 Sleep and movement differentiates actions of two types of somatostatin-expressing GABAergic interneuron in rat hippocampus. Neuron. 82:872–886. 24794095 10.1016/j.neuron.2014.04.007 PMC4041064

[BHV045C48] Kawaguchi Y Kubota Y 1997 GABAergic cell subtypes and their synaptic connections in rat frontal cortex. Cereb Cortex. 7:476–486. 9276173 10.1093/cercor/7.6.476

[BHV045C49] Kawaguchi Y Kubota Y 1998 Neurochemical features and synaptic connections of large physiologically-identified GABAergic cells in the rat frontal cortex. Neuroscience. 85:677–701. 9639265 10.1016/s0306-4522(97)00685-4

[BHV045C50] Kawaguchi Y Kubota Y 1996 Physiological and morphological identification of somatostatin- or vasoactive intestinal polypeptide-containing cells among GABAergic cell subtypes in rat frontal cortex. J Neurosci. 16:2701–2715. 8786446 10.1523/JNEUROSCI.16-08-02701.1996 PMC6578756

[BHV045C51] Kessaris N Magno L Rubin AN Oliveira MG 2014 Genetic programs controlling cortical interneuron fate. Curr Opin Neurobiol. 26:79–87. 24440413 10.1016/j.conb.2013.12.012 PMC4082532

[BHV045C52] Kisvarday ZF Adams CBT Smith AD 1986 Synaptic connections of axo-axonic (chandelier) cells in human epileptic temporal cortex. Neuroscience. 19:1179–1186. 3029627 10.1016/0306-4522(86)90131-4

[BHV045C53] Kisvarday ZF Gulyas A Beroukas D North JB Chubb IW Somogyi P 1990 Synapses, axonal and dendritic patterns of GABA-immunoreactive neurons in human cerebral cortex. Brain. 113:793–812. 2194628 10.1093/brain/113.3.793

[BHV045C54] Kubota Y 2014 Untangling GABAergic wiring in the cortical microcircuit. Curr Opin Neurobiol. 26:7–14. 24650498 10.1016/j.conb.2013.10.003

[BHV045C55] Kubota Y Shigematsu N Karube F Sekigawa A Kato S Yamaguchi N Hirai Y Morishima M Kawaguchi Y 2011 Selective coexpression of multiple chemical markers defines discrete populations of neocortical GABAergic neurons. Cereb Cortex. 21:1803–1817. 21220766 10.1093/cercor/bhq252

[BHV045C56] Letinic K Zoncu R Rakic P 2002 Origin of GABAergic neurons in the human neocortex. Nature. 417:645–649. 12050665 10.1038/nature00779

[BHV045C57] Lewis DA 2014 Inhibitory neurons in human cortical circuits: substrate for cognitive dysfunction in schizophrenia. Curr Opin Neurobiol. 26:22–26. 24650500 10.1016/j.conb.2013.11.003 PMC4024332

[BHV045C58] Liodis P Denaxa M Grigoriou M Akufo-Addo C Yanagawa Y Pachnis V 2007 Lhx6 activity is required for the normal migration and specification of cortical interneuron subtypes. J Neurosci. 27:3078–3089. 17376969 10.1523/JNEUROSCI.3055-06.2007 PMC6672459

[BHV045C59] Lovett-Barron M Losonczy A 2014 Behavioral consequences of GABAergic neuronal diversity. Curr Opin Neurobiol. 26:27–33. 24650501 10.1016/j.conb.2013.11.002

[BHV045C60] Ma T Wang C Wang L Zhou X Tian M Zhang Q Zhang Y Li J Liu Z Cai Y 2013 Subcortical origins of human and monkey neocortical interneurons. Nat Neurosci. 16:1588–1597. 24097041 10.1038/nn.3536

[BHV045C61] Ma T Zhang Q Cai Y You Y Rubenstein JL Yang Z 2012 A subpopulation of dorsal lateral/caudal ganglionic eminence-derived neocortical interneurons expresses the transcription factor Sp8. Cereb Cortex. 22:2120–2130. 22021915 10.1093/cercor/bhr296

[BHV045C62] Marin O 2012 Interneuron dysfunction in psychiatric disorders. Nat Rev Neurosci. 13:107–120. 22251963 10.1038/nrn3155

[BHV045C63] Marin O Muller U 2014 Lineage origins of GABAergic versus glutamatergic neurons in the neocortex. Curr Opin Neurobiol. 26:132–141. 24549207 10.1016/j.conb.2014.01.015 PMC4159607

[BHV045C64] Marin-Padilla M 1998 Cajal-Retzius cells and the development of the neocortex. Trends Neurosci. 21:64–71. 9498301 10.1016/s0166-2236(97)01164-8

[BHV045C65] Marin-Padilla M 1987 The chandelier cell of the human visual cortex: a Golgi study. J Comp Neurol. 256:61–70. 2434536 10.1002/cne.902560106

[BHV045C66] Marmigere F Ernfors P 2007 Specification and connectivity of neuronal subtypes in the sensory lineage. Nat Rev Neurosci. 8:114–127. 17237804 10.1038/nrn2057

[BHV045C67] Martinez-Cerdeno V Clasca F 2002 Reelin immunoreactivity in the adult neocortex: a comparative study in rodents, carnivores, and non-human primates. Brain Res Bull. 57:485–488. 11923015 10.1016/s0361-9230(01)00718-3

[BHV045C68] Meskenaite V 1997 Calretinin-immunoreactive local circuit neurons in area 17 of the cynomolgus monkey, *Macaca fascicularis*. J Comp Neurol. 379:113–132. 9057116

[BHV045C69] Meyer G 1983 Axonal patterns and topography of short-axon neurons in visual areas 17, 18, and 19 of the cat. J Comp Neurol. 220:405–438. 6643736 10.1002/cne.902200405

[BHV045C70] Meyer G Wahle P 1988 Early postnatal development of cholecystokinin-immunoreactive structures in the visual cortex of the cat. J Comp Neurol. 276:360–386. 3192767 10.1002/cne.902760304

[BHV045C71] Miyoshi G Machold RP Fishell G 2013 Specification of GABAergic neocortical interneurons. In: Kageyama R Yamamori T, editors. Cortical development: neural diversity and neocortical organization. Japan: Springer p. 89–126.

[BHV045C72] Molnar G Farago N Kocsis AK Rozsa M Lovas S Boldog E Baldi R Csajbok E Gardi J Puskas LG 2014 GABAergic neurogliaform cells represent local sources of insulin in the cerebral cortex. J Neurosci. 34:1133–1137. 24453306 10.1523/JNEUROSCI.4082-13.2014 PMC6705313

[BHV045C73] Molnar G Olah S Komlosi G Fule M Szabadics J Varga C Barzo P Tamas G 2008 Complex events initiated by individual spikes in the human cerebral cortex. PLoS Biol. 6:1842–1849. 10.1371/journal.pbio.0060222 PMC2528052 18767905

[BHV045C74] Monyer H Markram H 2004 Intemeuron diversity series: molecular and genetic tools to study GABAergic interneuron diversity and function. Trends Neurosci. 27:90–97. 15102488 10.1016/j.tins.2003.12.008

[BHV045C75] Morino P Mascagni F McDonald A Hokfelt T 1994 Cholecystokinin corticostriatal pathway in the rat: evidence for bilateral origin from medial prefrontal cortical areas. Neuroscience. 59:939–952. 7520138 10.1016/0306-4522(94)90297-6

[BHV045C76] Nery S Fishell G Corbin JG 2002 The caudal ganglionic eminence is a source of distinct cortical and subcortical cell populations. Nat Neurosci. 5:1279–1287. 12411960 10.1038/nn971

[BHV045C77] Ogawa M Miyata T Nakajima K Yagyu K Seike M Ikenaka K Yamamoto H Mikoshiba K 1995 The reeler gene-associated antigen on Cajal-Retzius neurons is a crucial molecule for laminar organization of cortical neurons. Neuron. 14:899–912. 7748558 10.1016/0896-6273(95)90329-1

[BHV045C78] Olah S Komlosi G Szabadics J Varga C Toth E Barzo P Tamas G 2007 Output of neurogliaform cells to various neuron types in the human and rat cerebral cortex. Front Neural Circuits. 1:4. 18946546 10.3389/neuro.04.004.2007 PMC2526278

[BHV045C79] Packer AM McConnell DJ Fino E Yuste R 2013 Axo-dendritic overlap and laminar projection can explain interneuron connectivity to pyramidal cells. Cereb Cortex. 23:2790–2802. 22941716 10.1093/cercor/bhs210 PMC3968298

[BHV045C80] Pesold C Impagnatiello F Pisu MG Uzunov DP Costa E Guidotti A Caruncho HJ 1998 Reelin is preferentially expressed in neurons synthesizing gamma-aminobutyric acid in cortex and hippocampus of adult rats. Proc Natl Acad Sci USA. 95:3221–3226. 9501244 10.1073/pnas.95.6.3221 PMC19723

[BHV045C81] Petanjek Z Berger B Esclapez M 2009 Origins of cortical GABAergic neurons in the cynomolgus monkey. Cereb Cortex. 19:249–262. 18477686 10.1093/cercor/bhn078 PMC2638783

[BHV045C82] Petersen C 2014 Cell-type specific function of GABAergic neurons in layers 2 and 3 of mouse barrel cortex. Curr Opin Neurobiol. 26:1–6. 24650497 10.1016/j.conb.2013.10.004

[BHV045C83] Pfeffer CK Xue M He M Huang ZJ Scanziani M 2013 Inhibition of inhibition in visual cortex: the logic of connections between molecularly distinct interneurons. Nat Neurosci. 16:1068–1076. 23817549 10.1038/nn.3446 PMC3729586

[BHV045C84] Pi H-J Hangya B Kvitsiani D Sanders JI Huang Z Kepecs A 2013 Cortical interneurons that specialize in disinhibitory control. Nature. 503:521–524. 24097352 10.1038/nature12676 PMC4017628

[BHV045C85] Pohlkamp T David C Cauli B Gallopin T Bouche E Karagiannis A May P Herz J Frotscher M Staiger JF 2014 Characterization and distribution of Reelin-positive interneuron subtypes in the rat barrel cortex. Cereb Cortex. 24:3046–3058. 23803971 10.1093/cercor/bht161 PMC4193467

[BHV045C123] Qin J Suh JM Kim BJ Yu CT Tanaka T Kodama T Tsai MJ Tsai SY 2007 The expression pattern of nuclear receptors during cerebellar development. Dev Dyn. 236:810–820. 17205580 10.1002/dvdy.21060

[BHV045C86] Quattrocolo G Maccaferri G 2014 Optogenetic activation of Cajal-Retzius cells reveals their glutamatergic output and a novel feedforward circuit in the developing mouse hippocampus. J Neurosci. 34:13018–13032. 25253849 10.1523/JNEUROSCI.1407-14.2014 PMC4172799

[BHV045C87] Radnikow G Feldmeyer D Lubke J 2002 Axonal projection, input and output synapses, and synaptic physiology of Cajal-Retzius cells in the developing rat neocortex. J Neurosci. 22:6908–6919. 12177189 10.1523/JNEUROSCI.22-16-06908.2002 PMC6757901

[BHV045C88] Rakic S Zecevic N 2003 Emerging complexity of layer I in human cerebral cortex. Cereb Cortex. 13:1072–1083. 12967924 10.1093/cercor/13.10.1072

[BHV045C89] Ramon y Cajal S 1899 Estudios sobre la corteza cerebral humana. Corteza Vis Rev Trim Microgr. 4:1–63.

[BHV045C90] Ramon y Cajal S 1911 Histologie du systeme nerveux de l'homme et des vertebres. Paris: Maloine.

[BHV045C91] Reinchisi G Ijichi K Glidden N Jakovcevski I Zecevic N 2012 COUP-TFII expressing interneurons in human fetal forebrain. Cereb Cortex. 22:2820–2830. 22178710 10.1093/cercor/bhr359 PMC3491767

[BHV045C92] Rogers JH 1992 Immunohistochemical markers in rat cortex: co-localization of calretinin and calbindin-D28k with neuropeptides and GABA. Brain Res. 587:147–157. 1356060 10.1016/0006-8993(92)91439-l

[BHV045C93] Rubenstein JLR Merzenich MM 2003 Model of autism: increased ratio of excitation/inhibition in key neural systems. Genes Brain Behav. 2:255–267. 14606691 10.1034/j.1601-183x.2003.00037.x PMC6748642

[BHV045C94] Rubin AN Kessaris N 2013 PROX1: a lineage tracer for cortical interneurons originating in the lateral/caudal ganglionic eminence and preoptic area. PLoS ONE. 8:e77339. 24155945 10.1371/journal.pone.0077339 PMC3796451

[BHV045C124] Schwaller B Brückner G Celio MR Hartig W 1999 A polyclonal goat antiserum against the calcium-binding protein calretinin is a versatile tool for various immunochemical techniques. J Neurosci Methods. 92:137–144. 10595711 10.1016/s0165-0270(99)00106-5

[BHV045C95] Somogyi P 2010 Hippocampus—intrinsic organisation. In: Shepherd GM Grillner S, editors. Handbook of brain microcircuits. Oxford: Oxford University Press p. 148–164.

[BHV045C96] Somogyi P 1977 A specific “axo-axonal” interneuron in the visual cortex of the rat. Brain Res. 136:345–350. 922488 10.1016/0006-8993(77)90808-3

[BHV045C97] Somogyi P Cowey A 1981 Combined Golgi and electron microscopic study on the synapses formed by double bouquet cells in the visual cortex of the cat and monkey. J Comp Neurol. 195:547–566. 7462443 10.1002/cne.901950402

[BHV045C98] Somogyi P Cowey A 1984 Double bouquet cells. New York: Plenum Press.

[BHV045C99] Somogyi P Freund TF Cowey A 1982 The axo-axonic interneuron in the cerebral cortex of the rat, cat and monkey. Neuroscience. 7:2577–2607. 7155343 10.1016/0306-4522(82)90086-0

[BHV045C100] Somogyi P Kisvarday ZF Martin KAC Whitteridge D 1983 Synaptic connections of morphologically identified and physiologically characterized large basket cells in the striate cortex of cat. Neuroscience. 10:261–294. 6633861 10.1016/0306-4522(83)90133-1

[BHV045C101] Somogyi P Takagi H 1982 A note on the use of picric acid-paraformaldehyde-glutaraldehyde fixative for correlated light and electron microscopic immunocytochemistry. Neuroscience. 7:1779–1783. 6181433 10.1016/0306-4522(82)90035-5

[BHV045C102] Szabadics J Tamas G Soltesz I 2007 Different transmitter transients underlie presynaptic cell type specificity of GABA_A_,slow and GABA_A_,fast. Proc Natl Acad Sci USA. 104:14831–14836. 17785408 10.1073/pnas.0707204104 PMC1964542

[BHV045C103] Szabadics J Varga C Molnar G Olah S Barzo P Tamas G 2006 Excitatory effect of GABAergic axo-axonic cells in cortical microcircuits. Science. 311:233–235. 16410524 10.1126/science.1121325

[BHV045C104] Szentagothai J 1973 Synaptology of the visual cortex. Berlin: Springer-.

[BHV045C105] Szentagothai J Arbib MA 1974 Conceptual models of neural organization. Neurosci Res Prog Bull. 12:305–510. 4437759

[BHV045C106] Tamas G Buhl EH Somogyi P 1997 Fast IPSPs elicited via multiple synaptic release sites by distinct types of GABAergic neurone in the cat visual cortex. J Physiol (Lond). 500:715–738. 9161987 10.1113/jphysiol.1997.sp022054 PMC1159420

[BHV045C107] Tamas G Lörincz A Simon A Szabadics S 2003 Identified sources and targets of slow inhibition in the neocortex. Science. 299:1902–1905. 12649485 10.1126/science.1082053

[BHV045C108] Thomson AM West DC Wang Y Bannister AP 2002 Synaptic connections and small circuits involving excitatory and inhibitory neurons in layers 2–5 of adult rat and cat neocortex: triple intracellular recordings and biocytin labelling in vitro. Cereb Cortex. 12:936–953. 12183393 10.1093/cercor/12.9.936

[BHV045C109] Toledo-Rodriguez M Blumenfeld B Wu C Luo J Attali B Goodman P Markram H 2004 Correlation maps allow neuronal electrical properties to be predicted from single-cell gene expression profiles in rat neocortex. Cereb Cortex. 14:1310–1327. 15192011 10.1093/cercor/bhh092

[BHV045C110] Toledo-Rodriguez M Goodman P Illic M Wu C Markram H 2005 Neuropeptide and calcium-binding protein gene expression profiles predict neuronal anatomical type in the juvenile rat. J Physiol. 567:401–413. 15946970 10.1113/jphysiol.2005.089250 PMC1474205

[BHV045C111] Tomioka R Okamoto K Furuta T Fujiyama F Iwasato T Yanagawa Y Obata K Kaneko T Tamamaki N 2005 Demonstration of long-range GABAergic connections distributed throughout the mouse neocortex. Eur J Neruosci. 21:1587–1600. 10.1111/j.1460-9568.2005.03989.x 15845086

[BHV045C112] Tricoire L Pelkey KA Daw MI Sousa VH Miyoshi G Jeffries B Cauli B Fishell G McBain CJ 2010 Common origins of hippocampal ivy and nitric oxide synthase expressing neurogliaform cells. J Neurosci. 30:2165–2176. 20147544 10.1523/JNEUROSCI.5123-09.2010 PMC2825142

[BHV045C113] Tripodi M Filosa A Armentano M Studer M 2004 The COUP-TF nuclear receptors regulate cell migration in the mammalian basal forebrain. Development. 131:6119–6129. 15548577 10.1242/dev.01530

[BHV045C114] Tsai SY Tsai MJ 1997 Chick ovalbumin upstream promoter-transcription factors (COUP-TFs): coming of age. Endocr Rev. 18:229–240. 9101138 10.1210/edrv.18.2.0294

[BHV045C115] Uematsu M Hirai Y Karube F Ebihara S Kato M Abe K Obata K Yoshida S Hirabayashi M Yanagawa Y 2008 Quantitative chemical composition of cortical GABAergic neurons revealed in transgenic venus-expressing rats. Cereb Cortex. 18:315–330. 17517679 10.1093/cercor/bhm056

[BHV045C116] Vucurovic K Gallopin T Ferezou I Rancillac A Chameau P van Hooft JA Geoffroy H Monyer H Rossier J Vitalis T 2010 Serotonin 3A receptor subtype as an early and protracted marker of cortical interneuron subpopulations. Cereb Cortex. 20:2333–2347. 20083553 10.1093/cercor/bhp310 PMC2936799

[BHV045C117] Wonders CP Anderson SA 2006 The origin and specification of cortical interneurons. Nat Rev Neurosci. 7:687–696. 16883309 10.1038/nrn1954

[BHV045C118] Xu Q Cobos I De La Cruz E Rubenstein JL Anderson SA 2004 Origins of cortical interneuron subtypes. J Neurosci. 24:2612–2622. 15028753 10.1523/JNEUROSCI.5667-03.2004 PMC6729522

[BHV045C119] Xu XM Roby KD Callaway EM 2006 Mouse cortical inhibitory neuron type that coexpresses somatostatin and calretinin. J Comp Neurol. 499:144–160. 16958092 10.1002/cne.21101

[BHV045C120] You LR Lin FJ Lee CT DeMayo FJ Tsai MJ Tsai SY 2005 Suppression of Notch signalling by the COUP-TFII transcription factor regulates vein identity. Nature. 435:98–104. 15875024 10.1038/nature03511

[BHV045C121] Zeng H Shen EH Hohmann JG Oh SW Bernard A Royall JJ Glattfelder KJ Sunkin SM Morris JA Guillozet-Bongaarts AL 2012 Large-scale cellular-resolution gene profiling in human neocortex reveals species-specific molecular signatures. Cell. 149:483–496. 22500809 10.1016/j.cell.2012.02.052 PMC3328777

